# 3D models of dilated cardiomyopathy: Shaping the chemical, physical and topographical properties of biomaterials to mimic the cardiac extracellular matrix

**DOI:** 10.1016/j.bioactmat.2021.05.040

**Published:** 2021-06-10

**Authors:** Marie Camman, Pierre Joanne, Onnik Agbulut, Christophe Hélary

**Affiliations:** a Sorbonne Université, CNRS, UMR 7574, Laboratoire de Chimie de la Matière Condensée de Paris, 4 place Jussieu (case 174), F-75005, Paris, France; b Sorbonne Université, Institut de Biologie Paris-Seine (IBPS), CNRS UMR 8256, Inserm ERL U1164, Biological Adaptation and Ageing, 7 quai St-Bernard (case 256), F-75005, Paris, France

**Keywords:** Biomaterials, Dilated cardiomyopathies, Induced pluripotent stem cells, Porosity, Anisotropy

## Abstract

The pathophysiology of dilated cardiomyopathy (DCM), one major cause of heart failure, is characterized by the dilation of the heart but remains poorly understood because of the lack of adequate *in vitro* models. Current 2D models do not allow for the 3D organotypic organization of cardiomyocytes and do not reproduce the ECM perturbations. In this review, the different strategies to mimic the chemical, physical and topographical properties of the cardiac tissue affected by DCM are presented. The advantages and drawbacks of techniques generating anisotropy required for the cardiomyocytes alignment are discussed. In addition, the different methods creating macroporosity and favoring organotypic organization are compared. Besides, the advances in the induced pluripotent stem cells technology to generate cardiac cells from healthy or DCM patients will be described. Thanks to the biomaterial design, some features of the DCM extracellular matrix such as stiffness, porosity, topography or chemical changes can impact the cardiomyocytes function *in vitro* and increase their maturation. By mimicking the affected heart, both at the cellular and at the tissue level, 3D models will enable a better understanding of the pathology and favor the discovery of novel therapies.

## Highlights

• Crucial need of 3D models of genetic dilated cardiomyopathies (DCM).
• 3D models allow a better understanding of the pathology and favor the discovery of new therapies.
• iPSC technology relevant to generate cardiomyocytes (iPSC-CMs) from DCM patients.
• The ideal scaffold has to be macroporous and anisotropic to promote iPSC-CMs maturation.
• 3D printing is a promising technique to design scaffolds mimicking the cardiac ECM.

## Graphical abstract

**Figure:**
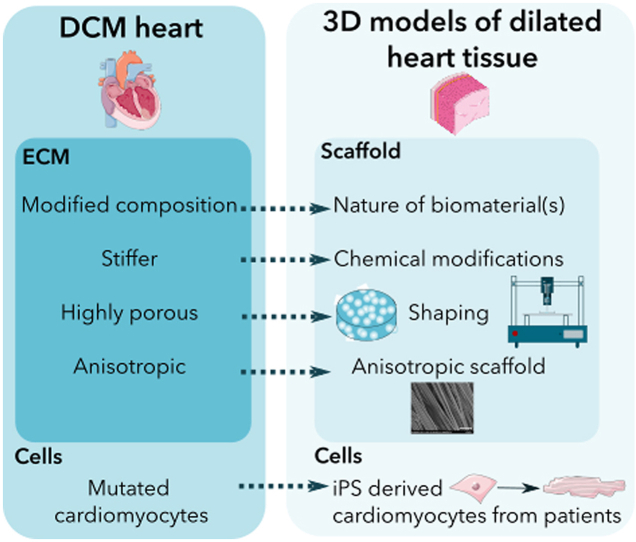

## 1 Introduction

The heart is a vital and complex organ which generates the blood flow through arteries and veins to irrigate organs in nutrients and oxygen. This organ also plays a crucial part in the control of blood pressure. The primary function of the heart resides in its contraction/relaxation cycles which are under a fine physiological regulation. When the cardiac output and/or the blood pressure in cardiac chambers are chronically out of physiological range, a heart failure occurs which eventually leads to death. Heart failure is one of the major causes of death worldwide as it affects approximately 1–2% of the global adult population in industrialized countries [1]. Among the different etiologies that are responsible for the development of heart failure, cardiomyopathies are one of the leading causes besides artery diseases and hypertension [2,3]. Cardiomyopathies are a heterogeneous category of cardiac pathologies characterized by anatomic and/or electrical dysfunction of the heart tissue. Among them, dilated cardiomyopathy (DCM) is the most common form and is known as one of the most frequent causes of heart failure with reduced ejection fraction [3]. Etiologies of DCM include genetic causes, infection or toxic agents, immune or metabolic diseases and cardiomyopathy associated with pregnancy (peripartum cardiomyopathy). Approximately 40% of diagnosed DCM cases originate from genetic causes, and their value is probably underestimated because of the heterogeneity of clinical symptoms [4]. Mutations in more than 100 genes have been reported to be involved in the development of DCM [5]. These genes are mainly involved in the force generation or transmission, the mechanosensing and the structural integrity of the cardiomyocytes, the cardiac contractile cells. For example, the gene encoding the giant protein titin, component of the sarcomere, is mutated in approximately 20–25% of DCM patients [6]. Mutations of other proteins, such as dystrophin, desmin or lamin A/C, are also associated with DCM.

As the clinical management of genetic-driven DCM is not effective for curing these diseases, there is an urgent need to develop pertinent models to better understand the pathophysiological mechanisms of DCM and to develop new treatments. Animal models of DCM were used to unravel the physiological mechanisms that underlie DCM [7]. Despite their great usefulness for fundamental research, the animal models appeared to be limited due to fundamental differences between animals and humans. This is especially the case in the field of early pharmacological drug development for which the need of simpler but pertinent model remains to be met. With the advent of the induced pluripotent stem cell (iPS) technology in 2007 [8], cardiomyocytes can be easily derived from patients affected by genetic-driven DCM. This technique allows the production of functional cells at any time by differentiation from iPS cells. Despite several drawbacks in terms of maturity, these functional cells are broadly used to model DCM *in vitro*. Besides the development of cellular models, biomaterials appear more and more suitable for cell culture as they aim at mimicking the physiological or the diseased extracellular matrix (ECM).

The most advanced 3D engineered heart tissue is a cellularized hydrogel consisting of primary cardiac cells and a mixture of collagen I and fibrinogen. Prior to gelling, the mixture is cast between two PDMS (polydimethylsiloxane) pillars to allow hydrogel attachment. This set-up is adequate for cell contraction studies [9] because it is closer to the physiological heart than the 2D cell layers. Streckfuss-Bömeke and co-workers measured an impaired force generation in diseased microtissues generated by a similar method [10]. However, this system suffers from other drawbacks which limit its utilization as cardiac model. First, hydrogels do not possess the suitable stiffness as the collagen concentration used is very low. In addition, the system evolves during the time course of the experiment because the cardiomyocytes contract and rearrange the collagen network. Last, this system is not porous.

In this review, we first provide an overview on the current models of DCM. Then, the different strategies to improve these models are presented. For this purpose, we focus on the biomaterial design to obtain the most relevant artificial extracellular matrix and the recent technologies to promote cardiomyocyte differentiation and maturation from iPS. Therefore, the different scaffolds mimicking the healthy and DCM cardiac extracellular matrix will be highlighted. Last, an overview of the different techniques suitable to assess the contractility of the engineered tissues will be presented.

## 2 Anatomical and cellular features of dilated cardiomyopathy

Structural modifications are visible at different scales of the heart. At the cellular level, cardiomyocytes have a lower force contraction which leads to an impaired ejection fraction of the blood in the circulatory system. Before the major symptoms appear, patients can be asymptomatic thanks to compensatory mechanisms through an increase of vasoconstriction and/or heart rate to maintain the physiological blood pressure. These compensatory mechanisms are associated with heart remodeling which finally becomes detrimental and irreversible in the long-term. A cellular hypertrophy is observed which partially compensates the loss of cardiomyocytes [11] (See Table 1). When the first symptoms occur, the major structural modification is the enlargement of the left ventricle, up to around 140% its initial size [12]. This enlarged ventricle has a thinner wall and weaker contraction abilities [13]. A healthy heart is able to pump 5 L of blood every minute, *i.e.* around 100 mL per beating, while in DCM, each beat ejects less than 65 mL^6^. When heart does not provide the demand in blood and oxygen for tissues, the patient develops a heart failure characterized by breathlessness and chest pain. Depending on the mutation, patients have different lifespans, but, after 60 years, all are affected by a severe heart failure that may cause a heart attack at any time.

**Table 1:** Biophysical comparison of a healthy and a dilated human heart.

Level	Parameters	Healthy heart	Dilated heart
Organ	Morphology	Left ventricle slightly larger than the right ventricle	Enlarged and weakened left ventricle [13]
		Diameter between 39 and 59 mm [12]	Diameter larger than 69 mm [12]
		
	Function	Full ejection fraction around 100 mL per beat [30]	Ejection fraction reduced of 45% [6]
			Not enough oxygen provided
		
	Remodeling	Reversible remodeling with endurance [15] or pregnancy	Irreversible remodeling: increased ECM deposition, fibrosis, inflammation [15]

Tissue	Stiffness	From 7 kPa to 15 kPa [31]	Increased due to fibrosis and collagen deposition [32]
			Decreased elasticity [33]
		
	Inflammation	Inflammation is due to infection, oxidative stress or hypertension [18,34]	Robust proinflammatory response following cellular damages [18]
			Recruitment of immune cells
		
	ECM	Fibers of collagen I and III (ratio 7:1) [35]	Overproduction of fibronectin and collagen [14] (especially I and VI [33]) Fibrosis occupy 20% of the volume [36]
		Proteoglycans	Loss of CM alignment
		Anisotropy to allow CM alignment	
		
	Capillary density	2439 ± 59 capillaries per mm [2,37]	1245 ± 345 capillaries per mm^2^
			Increased intercapillary distance [38]
			Shorter and smaller coronary arteries [22]
		
	Cell composition	Cardiomyocytes (18%), endothelial cells (24%) and mesenchymal cells (58%) [19] CM fill 75% of the volume [20]	Loss of 10% of CM [36] Decreased cell density

Cardiomyocytes (CM)	Sarcomeric passive tension	Ratio 30/70 between the two isoforms of titin	Reduced ratio 50/50 when titin is mutated [39]
			Faster relaxation kinetics [25]
			Myofibrils stiffness decreased by 26% [23]
		
	Contractile function	ECM stiffness for best force contraction generation [26]	Decreased force capacity depending on the mutation [23]
		
	Ionic exchanges	Calcium concentration is high during systole and low during diastole (from 1 μM to 100 nM) [40]	Increased calcium sensitivity, enhances the contraction rate [23] Tissue acidification alters electrical conduction [29]
		
	Cell metabolism	Adult cells consume fatty acids [41]	Metabolism switch to glucose consumption [27,42]
		
	Cell density & morphology	10^8^ cells/cm^3^	Loss of cardiomyocytes [17] Visible remodeling scars [43] Cell hypertrophy around 2 fold [36]
		High length/width ratio (7:1)	

The anatomical and functional alterations are the consequence of changes at the tissue level. The microenvironment of each cell is disturbed on several aspects: mechanical, biochemical and structural. In a healthy heart, the ECM production ensures a constant renewal but in the DCM heart, an overproduction of ECM induces a fibrosis [14]. Due to the excess of ECM deposition [15], cardiac tissue is stiffer and thinner causing cellular damages such as necrosis and a severe inflammatory response [[16], [17], [18]]. This fall of the cardiomyocytes population in the heart leads to an imbalance between cardiomyocytes, fibroblasts and endothelial cells [19]. Despite cardiomyocytes accounting for less than 25% of the cells, they occupy 75% of the volume [20]. When cardiomyocytes die in the DCM heart, other cells such as fibroblasts proliferate and increase the fibrotic phenomenon, thus a vicious circle is formed. Moreover, the cardiac endotelialization is also disturbed with a defective vascularization and impaired angiogenesis [21]. The reduced expression of ß-catenin impacts activation, proliferation and migration of endothelial cells to create new vessels [22]. These changes result in a reduced perfusion with an inadequate blood flow for cardiomyocytes.

At the cell scale, all these consequences are due to a mutation on genes encoding for proteins components of the cytoskeleton. This mutation affects the contraction force of each individual cell [23]. To compensate for this lack of efficiency, cells tend to hypertrophy [24], beat faster and shorten their relaxation kinetics [25]. In a healthy heart, every parameter such as the stiffness is finely tuned to optimize cell contraction efficiency [26]. As cells beat faster in the DCM heart, they consume more energy and they will switch their fatty acid metabolism to a glucose consumption [27,28]. This metabolism hastens the production of energy. Ionic channels change their sensitivity to calcium that leads to an acidification of the tissue and a poorer electrical conduction [29].

In an *in vitro* DCM model, the cellular parameters will be followed to determine the severity of the disease, its evolution and the impact of drugs to slow down or reverse some effects of the disease.

## 3 Cellular *in vitro* cardiac models

### 3.1 Cell selection for a relevant model of heart tissue

To develop a physiological cardiac tissue model of DCM, cell selection is crucial. As cardiomyocytes are the cardiac beating cells, they must be used to develop a heart model. Unfortunately, they are differentiated cells unable to proliferate, thereby limiting their use *in vitro*. Over time, cardiomyocytes from neonatal rats [44,45], rabbits [46] or chickens [47] have been used. Once collected, these cells lose their cardiac abilities and, additionally, animal cells differ from human ones. Moreover, no satisfactory immortalized cell lines exist for cardiomyocytes [48,49] because they do not contract and proliferate (not physiological for a cardiomyocyte). Pluripotent stem cells of embryonic origin (hESCs) or reprogrammed from adult somatic cells (such as induced pluripotent stem cells, iPS) are promising alternatives as these cells can be chemically differentiated into cardiomyocytes.

Takahashi and Yamanaka developed a technique to reprogram differentiated cells into embryonic-like stem cells [8]. Yamanaka obtained the Medicine Nobel Prize in 2012 for this reprogramming technique using four distinct growth factors (Oct4, Sox2, KIf-4 and c-Myc). This outstanding discovery opened a new field of research since cardiomyocytes-derived iPS cells can be obtained within a month. Several techniques are used to generate cardiomyocytes from pluripotent stem cells but the most popular method is the time-dependent induction of signaling pathways thanks to CHIR99021 and Wnt inhibitor [50]. This method is considered the easiest one to perform and affords an optimal differentiation yield with around 85% of differentiated cardiomyocytes. An additional step of purification yields to a 95% of cardiomyocytes ratio. As an alternative, microenvironment can also induce specific cell differentiation triggered by the substrate stiffness [51,52]. A recent study shows that pluripotent stem cells co-cultured with differentiated cardiomyocytes differentiate into neo cardiomyocytes [53].

Concerning hESCs, ethics and regulatory rules are the major drawbacks for their utilization [54,55]. In addition, the unavoidable challenge is to obtain hESCs with a genotype of interest, especially when the role of a mutation and/or specific genetic background associated with DCM needs to be assessed.

**Figure 1:**
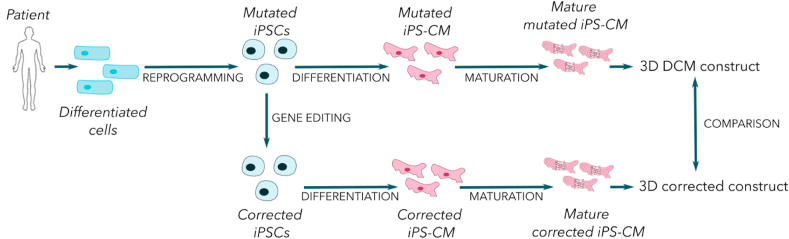
**Protocol to obtain iPS-CM derived from a patient affected by DCM.** This strategy allows a comparison between patient and healthy donor cells to have a better understanding of the disease and/or find new therapeutic approaches.

Nowadays, cardiomyocytes derived from induced pluripotent stem cells (iPS-CM) are the main source of cardiac cells used *in vitro* to study cardiomyopathies. Their main advantage is the ability to collect them from any patient, healthy or sick. Blood circulating cells [56] or dermal fibroblasts [57] are collected and reprogrammed *in vitro*. When a DCM is genetic, the cells express the disease features since they have the genetically inherited mutation. Such cells were used to mimic and study a specific pathology and to gain a better understanding of the specific features of pathologies such as Lamin A/C DCM [57] or titin DCM [56]. As these cellular models are derived from a specific patient, drug screening performed *in vitro* can lead to a personalized treatment [58]. To ensure the relevance of such a model, the cells can be genetically modified to correct the mutation by gene editing using the CRISPR-Cas9 technology. Thus, for each patient, two types of cardiomyocytes can be generated: diseased and corrected healthy cells [59,60] (Fig. 1) which is used as control. Discovered by Doudna and Charpentier, CRISPR-Cas9 are molecular scissors able to cut a piece of gene and to replace it with a designed DNA sequence. Recently, Rebs and co-workers used this gene editing technique to correct a DCM mutation in iPS cells from a patient and they succeeded in differentiating the corrected cells into cardiomyocytes [61].

One limitation of iPS-CM is their lack of proliferation. As they are differentiated, they have to be seeded with a high density to colonize the tissue. Physiological tissues are composed of 10^8^ cells per cm [3]. This cell density is difficult to reach *in vitro* and is costly and time-consuming. To overcome this limitation, two techniques are now available. The first one consists of *in situ* differentiation of undifferentiated cells able to proliferate inside the scaffold. The differentiation process will be triggered in a second time [62]. The second technique relies on a chemical combination to induce proliferation of non-mature cardiomyocytes which spontaneously differentiate. They succeeded in expanding massively iPS-CM until 100–250 fold was reached [63]. Another strategy is to reduce the size of the engineered tissue. Thanks to miniaturization, the number of cells required is much lower and it is easier to obtain a contiguous tissue [64].

iPS-CMs are subject to a major drawback: their lack of maturity [65]. iPS-CM express cardiac markers levels similar to those of fetal cardiomyocytes. The morphology and the structural organization are also immature: cells are not sufficiently elongated and their sarcomeres not well defined [66]. Fortunately, several strategies can be used to hasten cell maturation *in vitro*.

### 3.2 Current strategies to artificially mature iPS-CMs

**Fig. 2:**
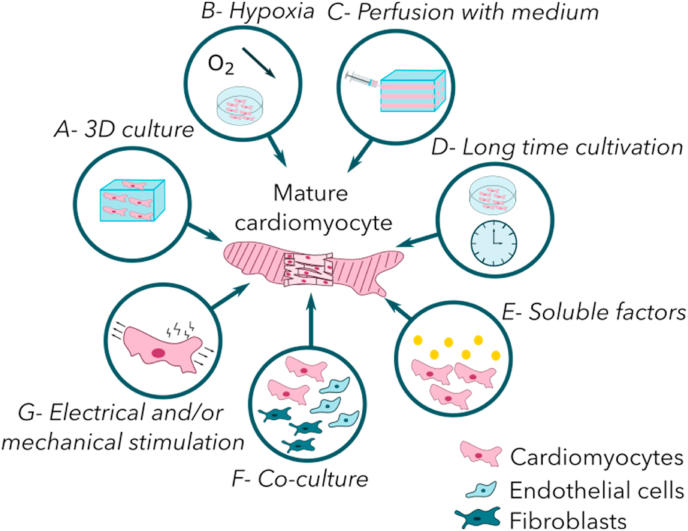
**Current strategies to mature cardiomyocytes *in vitro*** (Inspired from Jiang and al [67].). **A:** Moving to 3D culture is a key aspect of cardiomyocytes maturation because it reproduces the microenvironment of cardiac cells *in vivo*. **B:** Reducing oxygen level reproduces fetal conditions for cardiomyocytes. Cells are first conditioned in hypoxia and mature when the oxygen level rises. **C:** Perfusion with medium reproduces the blood flow in heart. This increases nutrient and oxygen diffusion, thereby promoting the cardiomyocyte viability. **D**: Long term cultivation is an obvious technique to age cardiomyocytes *in vitro*. **E**: Soluble factors such as hormones also enhance cell maturation as it reproduced the *in vivo* exposition to signaling molecules. **F**: Addition of other cell types reproduces the intercellular communications. **G:** A combination of electrical and mechanical stimulations trains the artificial tissue and synchronizes its beating.

To enhance cell maturation *in vitro*, several techniques must be combined to obtain an adequate maturation and mimic the cell microenvironment (Fig. 2).

Among these techniques, the simplest one consists in the long time cultivation to artificially age cardiomyocytes. This method gave mixed results with a higher expression of cardiac markers and a more physiological cell phenotype, only for a small fraction of cells [68]. However, most of these did not express these phenotypic markers. This technique is also limited by the high proliferative rate of non-myocyte cells such as fibroblasts that will become the predominant population if the cardiomyocyte purity is not high enough. Cardiomyocyte maturation can also be improved by the addition of growth factors into the culture medium. Angiogenic factors such as VEGF-A enhance vessel sprouting and cardiomyocyte maturation [69]. The addition of dexamethasone, a glucocorticoid hormone, alone [70,71] or combined with triiodothyronine [72] and insulin growth factor-1 (IGF-1) [20] can improve cardiomyocytes maturation. Cardiac markers and cell contractility are higher when these three biomolecules are combined. Oxytocin also influences cardiomyocyte differentiation and heart homeostasis [73]. Associated with female reproduction, this hormone plays a role in the healing process after infarction because of its anti-inflammatory and cardioprotective properties [74]. The addition of oxytocin into the culture medium improves the differentiation of embryonic stem cells into cardiomyocytes and induces cell contractility [75].

Another strategy focuses on cell metabolism. Immature cardiomyocytes use a glucose-based metabolism instead of a fatty acid one. A culture medium depleted in glucose encourages cardiomyocyte to switch to a fatty acid metabolism [76]. As a result, cardiomyocytes are more elongated and have more visible Z-lines as well as a higher expression of cardiac differentiation markers. The number of mitochondria per cell also increases as it is observed in adult cardiomyocytes. A change in oxygen level to mimic the *in utero* development of the heart is also a culture condition that can improve cell maturation. Hypoxic culture conditions lead to a higher ratio of cardiac differentiation and more mature cells [77]. Hypoxic conditions also tend to increase the expression of VEGF-A and the angiogenesis stimulation when cardiomyocytes are co-cultured with endothelial cells. An enhanced angiogenesis is beneficial to mature cardiomyocytes.

The microenvironment is a key element to ensure cell development. So, the use of a 3D scaffold to culture cells increases their maturation. With perfusion and sparse 3D structures, cells survived for 6 weeks [62] or even for as long as 98 days [78]. Medium perfusion in the structure [79] or porous substrates [80,81] allowed a uniform distribution of nutrients and oxygen, with a consequent high viability of cells in the whole structure being observed for a lengthy cultivation period. Cell survival was optimal regardless of its location, whether on channel walls or within a hydrogel [82]. Moreover, perfusion also mimics the blood pressure and induces the cardiomyocyte maturation by mechanical stimuli [83,84]. Blood pressure plays a significant role in contraction acting as a contraction trainer for cells thanks to fluid pressure [85]. Force contraction of the cardiomyocytes is twenty times higher after a week in perfused conditions than that of the static ones [86]. However, this contraction is not strong enough to mimic the physiological heart. The first studies using external mechanical stimulation of embedded cardiomyocytes in a collagen I/ECM protein gel [47,87] led to an increase of the force contraction and the calcium release transients [88]. It also induced cell hypertrophy and elongation, markers of cardiac maturation.

Cardiomyocytes are also sensitive to electrical stimulation; their beating rate is synchronous thanks to an electric wave. An electrical stimulation progressively extends the propagation of the electric wave on the entire structure [62] and cells beat synchronously. Moreover, electrical stimulation enhances cell ultrastructure: sarcomeres are more defined, gap junctions and intercalated disks are well developed [89]. Combining mechanical and electrical stimulation enhances cell alignment, maturation and hypertrophy [90] and induces a pace in the engineered heart tissue with higher force contraction. Sun and Nunes reviewed the different techniques to stimulate cells mechanically and electrically [91].

Combining several maturation techniques seems to be the best way to artificially mature iPS-CM [92]. Ronaldson-Bouchard and co-workers obtained iPS-CM with visible sarcomeres, gap junctions and functional calcium handlings [90] due to an electro-mechanical stimulation on a 3D construct. Once cells are mature enough, the 3D model of DCM can be used to understand the pathology or to test new drugs.

### 3.3 From the simplest approaches in 2D to the most complex developments of 3D cardiac models

**Fig. 3:**
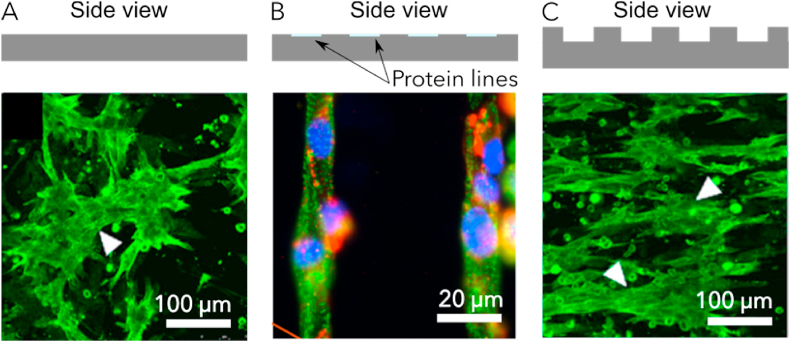
**Comparison of cardiomyocytes alignment depending on the substrate:** A- flat polysterene culture plate [97], B- patterned lines of laminin (non published results), C- nanogrooved substrate of polystyrene [97]. Compared to flat surface, cells align along the lines or the nanogrooves. Immunostaining: A and C: α-actinin, B: α-actinin (green), DAPI (blue) and Connexin 43 (red).

In the pharmaceutical industry, the evaluation drug toxicity or screening of novel molecules tested for cardiac therapies relies on 2D cell culture on plastic dishes (Fig. 3-A). More recently, models dedicated to a specific pathology have emerged through which more knowledge can be gained regarding the disease and its features. Sun and co-workers generated cardiac cells from iPS cells of DCM patients. They compared these cells with healthy cells from other members of the family, allowing an altered regulation of calcium transients and decreased contractility to be observed [93]. These results were similar to those obtained by Shah and co-workers even though the mutation was different [94]. These models provide for useful information regarding DCM pathophysiology but they lack physiological relevance as the cell behavior differs from that in 3D.

The main advantage of 2D cultures is their high reproducibility, and this justifies their broad use for pharmacological drug screenings. Morphology and contractility of cardiac cells can be easily analyzed owing to the unique layer of cells. However, they are far from DCM cardiac tissue in important respects: they do not possess a disturbed ECM with the specific stiffness, anisotropy and porosity. To improve 2D models, cardiomyocytes can be aligned using patterned lines of proteins [95] (Fig. 3-B) or nanogrooved substrates [96,97] (Fig. 3-C). The patterned lines are often generated by microstamping: a stamp with lines is impregnated with proteins and then stamped on a glass slide. Besides the patterns, the substrate is given a non-adhesive coating. Nanogrooved substrates are fabricated using photolithography. A silicium wafer is designed with the negative grooves and PDMS (Polydimethylsiloxane) is molded on top to obtain a grooved substrate. The mold is then coated with adhesion proteins. Patterned lines are chemical guides for cardiomyocytes whereas grooves provide topographical guidance. The cell alignment observed on micropatterns or within grooves restores the gap junctions and ions channels, thereby enabling an improved conduction of contraction between cells. Differences between healthy and mutated cells can be discerned at the cell scale by electrophysiological measurements.

DCM affects the microenvironment of cells *in vivo* and these conditions cannot be reproduced *in vitro* with 2D cultures. Over the last decade, 2D culture models have been progressively replaced by more realistic 3D ones. In a 3D environment, cells interact with each other by secreting soluble factors, feel the appropriate matrix stiffness and restore cell-cell/cell-ECM contacts. These 3D models seem to be more suitable to the reproduction of DCM *in vitro*. The use of ECM proteins mimics the biochemical microenvironment of *in vivo* cells [98] and confer a more physiological shape to the cells. The first proof of concept of cardiac 3D culture consists of chicken cardiac cells embedded in a collagen I/ECM protein matrix to form spheroids [47]. Cells beat and a mechanical stimulation reinforces them. The recent emergence of 3D bioprinting accelerates this technology and offers the ability to create more complex structures [99]. Computer-associated design of structure allows for the generation of all types of shapes and the printer produces these with a high fidelity regardless of its complexity. Computed tomography (CT) scans of healthy hearts are now used as models for bioprinting to mimic the heart shape and its vascular network [62,100].

Few systems use iPS-CM derived from DCM patients in a 3D engineered heart. Mostly, these systems are collagen-based gels in which iPS-CM are embedded. The biochemical composition of these models is relevant as the cardiac ECM mainly consists of collagen I. However, hydrogels produced *in vitro* are softer and less porous than the physiological ECM. Hinson and co-workers demonstrated the impaired contractility of DCM cardiomyocytes encapsulated within collagen hydrogels compared to healthy cells [56]. However, this system still needs improvement to increase its biomimetism as the matrix composition is far from the physiological one. Using the same method, Stillitano and co-workers showed lower contraction rates and impaired force generation in the mutant cells than those of the corrected cells [101](using the CRISPR/CAs 9 gene editing). By focusing on a different mutation, Streckfuss-Bömeke and co-workers developed a DCM cardiac model based on the same strategy and observed altered calcium transients when the gel was mechanically stretched [10]. In this type of model, cells are aligned due to the mechanical stretching but their stability over time is uncertain. Ma and co-workers used a different strategy with filamentous 3D matrices (made of a photo-curable organic-inorganic hybrid polymer OrmoClear®) with mutated and corrected iPS-CM. They demonstrated that mutated tissues have an impaired force contraction and abnormal calcium transients [102]. Even if this matrix possesses mechanical and topographical properties, its synthetic composition is not entirely relevant to mimic DCM matrix. Hence novel strategies are required to mimic biological, physical and topographical cues of DCM extracellular matrix.

3D models can be more sophisticated with the addition of other cell types present in the heart tissue. Adding fibroblasts [103], epicardial derived cells [104] or human cardiac stem cells [105] positively impacts the morphology of cardiomyocytes with an increased length/width ratio. Besides, the addition of endothelial cells promotes the structural organization of cardiomyocytes [82]. Taken together, these results show that the best strategy for cardiac tissue engineering is the combination of the three main cell types (fibroblasts, endothelial cells and cardiomyocytes) to improve the contractility, cardiac markers expression and the structural organization of cardiomyocytes. Huang and co-workers conducted experiments to optimize the ratio between fibroblasts, cardiomyocytes and endothelial cells into an ECM-based hydrogel. As fibroblasts actively proliferate, the culture conditions were tuned to preserve enough empty space for cardiomyocytes to enlarge until they filled 75% of tissue volume as seen *in vivo* [20]. However, adding other cell types increases the complexity of the model and a compromise must be established.

Despite many advances in biomaterials and in cardiomyocytes cultivation, the 3D models lack anisotropy and suitable mechanical properties. By investigating these two aspects of cell microenvironment in 3D, models could evolve toward more physiological mini hearts.

## 4 The ideal biomaterial to mimic DCM tissue

Scaffolds are key elements to mimic the architecture of the heart extracellular matrix. In DCM, the ECM is stiffer, more fibrous, with a lower porosity than that of the healthy heart. This is due to the excess of collagen deposition leading to fibrosis and capillary spacing. These matrix features need to be reproduced *in vitro* before the combination with DCM cardiomyocytes. First, the scaffold has to allow for cell adhesion and survival. Second, it has to reproduce the ECM biochemical composition and physical properties observed in DCM: stiffness [31], elasticity [106] and stability [107]. Third, an adequate topography must be created including porosity to promote efficient diffusion of nutrients and oxygen and anisotropy to induce cell alignment [108,109].

### 4.1 Mimicking biochemical cues of cardiac ECM

Biochemical characteristics of biomaterials have to fit with the organ features they aim to reproduce and they have to allow cell culture [31]. Cardiac ECM is mainly composed of aligned collagen I fibrils around a very porous structure. Hence, the ideal biomaterial has to mimic this biochemical composition and topography.

#### *4.1.1* **Natural polymers**

Natural polymers are biodegradable, biocompatible and promote cell adhesion. Collagen [99,110], hyaluronic acid [111], gelatin [80], alginate [44], cellulose [112], fibrin [113] or chitosan [45] are biopolymers suitable for the fabrication of tissue engineered scaffolds. Collected from animals or plants, the physiological structure observed *in vivo* is difficult to reproduce *in vitro*. As a consequence, this lack of biomimetism results in poor mechanical properties. Collagen I is the main constituent of the physiological cardiac ECM and positively impacts the cardiomyocyte differentiation [114] by upregulating the gene expression of sarcomeric markers such as troponin [115]. Currently used at a low concentration, collagen hydrogels are too soft to mimic physical properties of heart ECM. Several strategies have been developed to increase the hydrogel stiffness such as cross-linking, use of highly concentrated solutions or combination with other polymers. For instance, using a collagen concentration at 20–40 mg mL^−1^ increases the hydrogel mechanical properties up to the values measured *in vivo* for the cardiac ECM [116,117]. In addition, a mix of highly concentrated collagen I and ECM powder is promising to reproduce the complexity of *in vivo* cardiac ECM.

Gelatin may also be used to form a hydrogel but this denaturated form of collagen does not reproduce cell-ECM interactions [80]. Associated with chitosan [118] or ECM proteins [119], gelatin has a lower degradation kinetics, better mechanical properties and cell-ECM interactions.

Alginate and agarose are too soft to reproduce cardiac ECM alone, but they become suitable for cardiac biomaterials after the addition of cross-linkers. Several crosslinkers exist such as glutaraldehyde [120], riboflavin/UV light [121], genipin [122] and carbodiimide [123]. However, these are not natural components of the cardiac ECM, so their utilization for a biomimetic approach is questionable. Moreover, the quantity of crosslinkers has to be adapted to prevent the cytotoxic effect.

To overcome their poor mechanical properties and stability, natural polymers such as collagen and gelatin can be functionalized by crosslinkable groups such as metacrylate [124]. Metacrylate functions polymerize to create a random mesh stiffer than the original mesh. Crosslinking of collagen I hydrogels lowers their swelling ability but increases their strength, stiffness and stability [125]. Thus, cross-linking is of particular value to model the fibrotic cardiac ECM characterized by a high stiffness and a low deformability.

In DCM, it is well known that fibroblasts overproduce specific proteins of extracellular matrix [14]. These ratios can be reproduced *in vitro* by mixing several natural biopolymers to mimic either a healthy matrix or a diseased one.

#### 4.1.2 Synthetic polymers

Synthetic materials made of polymers such as polycaprolactone (PCL) [126,127], polyethylene glycol (PEG) [128] or polyvinylidene fluoride (PVDF) [129] possess high and tunable mechanical properties. They were first used on their own to design scaffolds for cardiac engineering but are now combined with natural polymers. Usually, synthetic polymers are coated with ECM proteins (collagen [130], gelatin [126] or other ECM proteins) to improve their poor abilities for cell adhesion and spreading. The substrate becomes less hydrophobic and enables specific contacts with cellular receptors such as integrins. The main advantage in their utilization is their easy shaping and high reproducibility. Li and co-workers developed a cardiac tissue-like construct consisting of PLGA nanofibers by electrospinning to guide the growth of hiPSC-CMS. The nanofibers (diameter comprised between 500 nm and 2 μm) were designed to mimic collagen fibers in cardiac tissue. Because of this topography, the upregulation of cardiac biomarkers was observed and contractile function improved [131]. This study was performed with healthy hiPSC-CM but can easily be used to study DCM cardiomyocytes in order to analyze the changes in contractile function.

#### 4.1.3 Decellularized tissues

Decellularized matrix offers the best cell-ECM interactions [132] because it consists of a mix of natural proteins and proteoglycans that enhances cell adhesion and maturation. Decellularized matrices can be obtained from healthy or DCM hearts, although their availability is limited. All recognition domains used for cell attachment are present [133]. Using detergents, human pericardium isolated from cadavers is decellularized [118,134]. Because of the lack of human supplies, porcine myocardium is also generally used [119,133]. Lu and al. have decellularized a whole mouse heart and recellularized it with human iPS cells derived from cardiac progenitor cells [135]. Cells seeded on the construct differentiate into cardiomyocytes, endothelial cells and smooth muscle cells. Thanks to the addition of a perfusion system, cardiomyocytes beat for over 20 days in culture. Several techniques allow for tissue decellularization but the main challenges are the conservation of most of ECM components and the persistence of adequate mechanical properties [136]. Unfortunately, decellularized matrices lack mechanical stability (altered compared to native ECM), and a batch to batch variability is observed [137]. Nowadays, the utilization of ECM powder is preferred to complete decellularized matrices [118]. ECM powder possess(es) the same biochemical properties that enhance cell adhesion and orientate them towards cardiomyocyte maturation [135]. Combined with PEG hydrogels [138], the stiffness is enhanced due to the chemical cross-linking. This leads to a more suitable DCM extracellular matrix *in vitro* [47,110,139].

### 4.2 Tuning the mechanical properties of biomaterials to mimic DCM

A number of studies have investigated the cardiac stiffness *in vivo.* Several groups measured a Young's modulus of around 10 kPa in spite of variations during the contraction cycle. At the beginning of contraction, the stiffness is c.a 15 kPa but decreases to c.a 7 kPa at the end of the systolic phase [31]. This specific stiffness allows gap junctions between cells [106,109]. These junctions play a crucial role in contraction conduction and a change in stiffness severely affects the force contractility of cells as observed in DCM.

DCM is characterized by an excess of collagen deposition leading to the formation of a fibrotic tissue. As a consequence, the heart stiffness increases up to 35 kPa [140] thereby impairing the contraction conduction and cardiomyocytes viability. *In vitro*, the substrate stiffness impacts iPS-CM cultured on stiff polyacrylamide gels (120 kPa) with the aim of mimicking fibrotic tissues. The upregulation of cardiac-fibrosis-associated transcripts such as collagen I and III are observed on the substrates [141].

Thus, the mechanical properties of cardiac tissue models must be precisely tuned to reproduce *in vitro* the fibrotic DCM tissue.

### 4.3 3D shaping to model the DCM extracellular matrix

In a healthy heart, the myocardial capillary density is around 2400 capillaries per mm^2 37^ to ensure oxygen and nutrient diffusion to the cardiomyocytes, whereas in the DCM heart it decreases to 1245 capillaries per mm^238^. Because of cardiomyocyte hypertrophy in DCM, the intercapillary distance increases and impedes the diffusion of nutrients and oxygen in the tissue [38]. Capillaries are difficult to reproduce *in vitro* due to their small size and their complex network; however, porosity generation techniques that allow the physiological perfusion of oxygen and nutrients can be used. The modulation of the number of pores inside the biomaterial can be performed to reproduce the DCM vascular network.

Basically small pores (<20 μm in diameter) are dedicated to nutrient diffusion or angiogenesis and vasculogenesis when endothelial cells are added [142]. For cardiomyocytes cultivation, larger pores are required. A macroporosity with pores larger than 100 μm in diameter is adapted for the 3D development of cardiomyocytes and penetration [143,144]. This enables cardiomyocytes organotypic growth and promotes cell maturation owing to cell-cell interaction [80]. Another aspect of porous structures is the interconnectivity between pores, a parameter impacting cell colonization and survival in the scaffold which needs to be optimized (See Table 2).

**Table 2:** Comparison of the techniques to obtain a porous substrate suitable to cultivate cardiomyocytes.

Techniques	Self-assembly	Freeze casting	Porogen agent	3D printing	Needles
Size of the pores	100 nm–10 μm	Tunable (>10–100 μm)	Depends on the porogen agent (>100 μm)	Size of the printing head (>100 μm)	Size of the needles (>100 μm)

Geometry	Not controlled	Pores formed by ice crystals	Crystal shape	Channels	Channels

Interconnectivity	Not controlled	Not controlled	Not controlled	Controlled	Not controlled

Position in the material	Not controlled	Not controlled	Not controlled	Controlled	Controlled

Easy to handle	No	Yes	Yes	No	Yes

Nutrient diffusion	Yes	Yes	Yes	Yes	Yes

Cardiomyocytes organotypic growth	No	Yes	No	Yes	Yes

Easy to perfuse/vascularize	No	Yes	No	Yes	Yes

References	[[149], [150], [151]]	[153]	[160]	[62,100]	[161]

#### 4.3.1 Molded biomaterials

A simple approach relies on molding the substrate in the wells of a culture plate or in a PDMS mold to form hydrogels [9,145]. For instance, Masumoto and co-workers used collagen I mixed with Matrigel® and human iPSCs-CM to form an engineered heart *in situ* [146]. This technique does not create pores but the final volume of the construct is small enough to ensure nutrient diffusion and prevent necrotic core formation. UV-light, heating above 37 °C or chemicals are harmful to cells so the gelation process must avoid these cross-linking techniques to preserve cells. As collagen and Matrigel® do not require heating above 37 °C or UV light for gelling, cells bear the encapsulation process inside the gel. Cast hydrogels often do not mimic the natural microenvironment of cells because there is no vasculature or pores.

To generate porosity within a plain scaffold, laser ablating techniques have been used. This technique is based on the focusing of a laser beam that will induce local dissolution of hydrogel to form a desired shape and porosity. This process is very accurate, depending on the laser used, and the pore size could be adjusted to suit particular applications in tissue engineering such as neuronal guidance [147]. Unfortunately, the channel diameters are around 10 μm which is not large enough for cardiac cell organotypic growth.

#### 4.3.2 Techniques used in chemistry and material science to create porosity

##### 4.3.2.1 Self-assembly of polymers

**Fig. 4:**
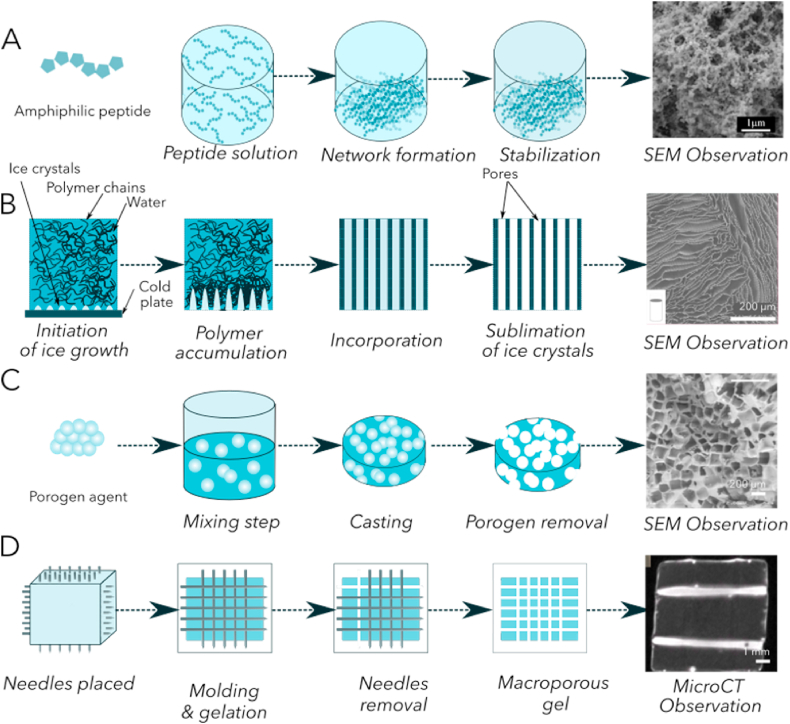
**Strategies to create pores of specific diameters.** The diameter of pores is designed for a specific application: to enhance nutrient diffusion, to improve cell colonization or to create a vascular network. A- Self-assembly peptides to create a nanoporous mesh with homogeneous pores (scale bar 1 m) [163].Cardiomyocytes do not penetrate, they must be encapsulated prior to gelation. B- Freeze-casting applied to a polymeric solution to create anisotropic pores with a controlled diameter [153]. Pores are large enough for cardiomyocytes culture (scale bar is 1 µm). C- Porogen agent removal, macroporous network shaped by the porogen agent [164]. D- Needles molding (same principle with nylon fibers) and removal after gelation. Large pores suitable for 3D organotypic growth of cardiomyocytes (non published results).

Self-assembly is the spontaneous organization of molecules into an ordered structure [148](Fig. 4-A). The pores are homogeneous and well distributed within the gel but they remain small. Amphiphilic peptides are good candidates for self-assembly because their negatively charged part creates non-covalent bonds with the positively charged side of another peptide. Schneider and co-workers synthesized a 16 residue peptide able to form stable sheets in water. The gelation process is tuned with temperature, pH and increasing salt concentration [149]. The gel was grafted and was easily colonized by host cells that suggests a good cytocompatibility and an easy penetration of cells. However, this is a microporosity rather than a macroporosity since the pores are around 10 μm in diameter. Hence it, is only suited to the diffusion of nutrients.

Rexeisen and co-workers developed a fibronectin mimetic peptide sequence with amphiphilic properties to induce self-assembly. This sequence contains an RGD domain to promote cell adhesion and another domain called PHSRN known to enhance cell interactions. This peptide naturally forms nanofibers in solution and gelifies. Unfortunately, the Young's modulus measured is around 500 Pa, i.e. very low compared to the cardiac ECM [150]. Ban and co-workers observed a 90% cell survival after 7 days in culture for neonatal murine cardiomyocytes encapsulated within this nanomatrix [151]. However, this technique generates pores that are too small to allow for cell assembling in an organotypic structure.

##### 4.3.2.2 Freeze-casting

The freeze-casting or ice templating is a process using a progressive decrease in temperature with a specific rate to induce a directional ice crystal formation inside a polymer solution (Fig. 4-B). Polymers such as alginate [152], collagen [153] or silk protein [154] are first dissolved in water to form a solution of the desired concentration. Ice growth within the solution can be random, with no specific direction; radial, crystals grow from the perimeter to the center or axial; crystals grow along one axis. Once ice crystals are formed, the polymer is cross linked or gelified, then freeze dried to remove water. Control of the cooling rate allows for tuning of the pore size. The high porosity of freeze casting constructs reduces their mechanical properties but collagen I scaffolds shaped by freeze-casting have straight pores able to align murine myoblasts [153]. This characteristic pore size of around 100 μm allows cardiomyocytes colonization.

##### 4.3.2.3 Porogen agent

The removal of a porogen agent such as salt, wax or sugar crystals encapsulated within the polymeric scaffold can create pores [155](Fig. 4-C). Porogen agents are first mixed with the polymer solution and dissolved in a second time after scaffold formation. For instance, NaCl salt crystals can be mixed with PLA in a dichloromethane solution. Then, the scaffold is formed by solvent evaporation and the porosity generated by porogen dissolution. The matrix must be stable during this process to reveal pores with a high interconnectivity [156]. The major drawback of this process is due to the removal step of porogens which limits the scaffold thickness [157]. Using NaCl crystals, Tadic and co-workers generated pores from 250 to 400 μm in diameter with an interconnection larger than 10 μm. This structure of pores is well adapted for cell culture and efficient nutrient diffusion. Sucrose or sodium bicarbonates crystals create smaller pores from 125 to 250 μm in diameter [158]. Using a commercial polymer Vicry, Xu and Quin obtained high aspect ratio channels – 322 × 8000 μm – [159]. The challenge with this process is always the removal step.

Ganji and co-workers used a salt leaching technique to create a microporous substrate for cardiomyocytes. The microporosity enhances cell communication and improves the electrical stimulation efficiency [160]. However, this method does not allow for cell encapsulation within porosity as solvents are used in the process. Cardiomyocytes are seeded on top of the scaffold and have to migrate within to colonize it. Despite the high rate of porosity (90%), pores are not large enough to ensure cardiomyocytes organotypic organization.

#### 4.3.3 Needles (or fibers) molding

To generate macroporosity, needles can be placed during the polymer casting in mold and removed after gelation [161] to create straight channels (Fig. 4-D). This technique represents an easy and rapid way to form straight channels. It is mainly used to reproduce vessels and these are colonized by endothelial cells [142]. The same procedure can be performed using nylon fibers to create channels [162]. The large range of diameters available allows the formation of pores of different size (from 100 μm to 500 μm).

#### 4.3.4 3D printing to combine shaping and porosity generation

**Fig. 5:**
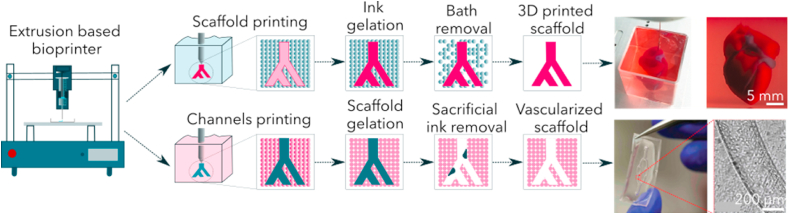
**3D printing to combine shaping and porosity. First line**: 3D printing in a support bath (Inspired from McCormack and al. [169]). The ink is printed in a support bath that allows printing of all complex shapes. After ink gelation or cross-linking, the support bath is removed by heating and reveals the final scaffold [100]. **Second line**: 3D printing in a cellularized scaffold. The sacrificial ink is printed within the cellularized scaffold and, after scaffold gelation, the sacrificial ink is removed by 1) heating; 2) solving; or 3) colding. The removal reveals porous channels [124].

Before the advent of 3D bioprinting, cells were seeded on top of hydrogels [44] or mixed with the polymer solution before gelling [146]. The emergence of bioprinting in the last decade has revolutionized the field since tissue shaping is now feasible. Bioprinting mainly uses an extrusion-based process [165] and resolution is limited by the needle diameter [166]. The ink is placed in a syringe and thanks to an x, y and z platform, it moves to extrude the ink and follow the pattern previously designed. A specific shape is developed on a computer and printed with the suitable bio-ink. To mimic heart morphology, X-ray tomography scans of patients are converted into 3D printed files and reduced to create mini hearts [62,100,167]. These complex shapes do not allow cells to survive if they are not vascularized [62]. This technique is illustrated on Fig. 5 line 1.

To mimic the heart vasculature, many scaffolds possess a network of small channels to increase nutrient diffusion. In addition, a larger macroporosity can be created to help cells to form organotypic structures and to mimic their 3D environment. With 3D printing, channels can be obtained by the use of a sacrificial ink removed after printing [168]. Basically, the bioprinter prints two different inks: the first extrusion head forms the biomaterial structure and a second one makes the sacrificial matrix. This latter is specifically chosen to be easily removable by 1) heating, 2) solvent addition or 3) cold exposition without impacting the scaffold structure and stability. After the printing and gelation step, the sacrificial ink is removed to reveal pores or channels. Many materials can be used as sacrificial inks, including, for example, gelatin which liquefies at 37 °C (1) [86,99] making it easy to remove. Organic solvent dissolves polycarbonate when it is used to create channels (2) [81] and a cold exposition liquefies Pluronic F127 and flushes it out (3) [82,127]. This removal step is performed only after the gelation step of the scaffold around the channels. The appropriate combination scaffold/sacrificial matrix is a key requirement because the removal of the second network (sacrificial matrix) must not alter the first one (scaffold).

Hinton et al. developed a promising approach employing 3D printing to recreate the complex structural properties of the heart using a macroporous scaffold. To support the printing of their complex shape, they used a support bath made of a thermo-reversible hydrogel such as gelatin spheres. Gelatin was stiff enough to support the printing of the construct performed at room temperature. At the end of the printing, the scaffold printed with an alginate bio-ink and a cross-linker was stiff enough to be heated to 37 °C. This step allows an easy removal of the support bath of gelatin through heating (Fig. 5 line 2). This enables the printing of the hydrated hydrogels with a low elastic modulus in a mechanically stable structure [167].

The different inks have to be biocompatible with a suitable viscosity to be printable [170]. Most studies use cellularized inks, called bio-inks, in which cells are directly mixed with the polymer solution in the printing head. The inks need to ensure cell survival as Gaetani and co-workers demonstrated using human cardiac progenitor cells in a solution of alginate that preserves their viability [171]. Alginate, gelatin, collagen I or decellularized ECM are often used as ink for bioprinting. For instance, Pati and co-workers developed an ink made from decellularized heart tissue which preserves critical components of the cardiac ECM [172]. Using bio-inks, cells are seeded with high precision and a uniform distribution within the scaffold. The challenge of *in vitro* heart tissue engineering is to increase the complexity of the environment of cardiomyocytes compared to the basic 2D cell culture while maintaining sufficient control in reproducibility. The 3D printing techniques comply with specifications since the shape is precisely designed on a computer and the printer follows exactly the same procedure each time.

### 4.4 Topographical relevance with an anisotropic scaffold

**Fig. 6:**
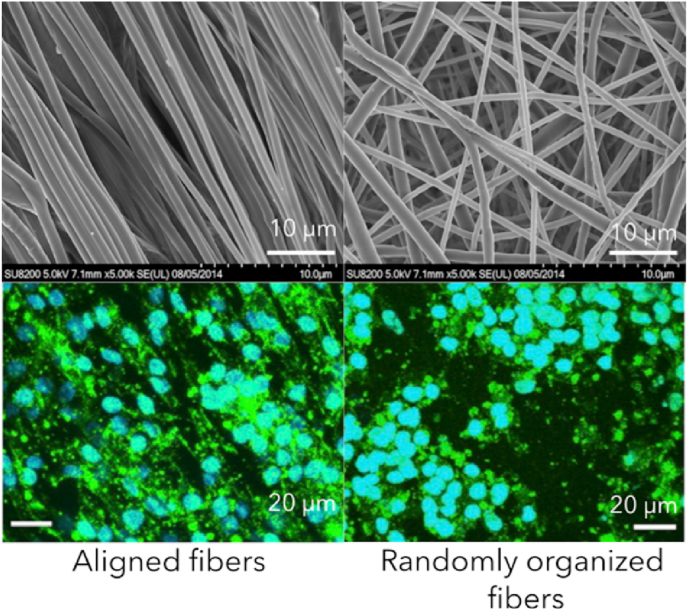
**Cardiomyocytes alignment on anisotropic substrate** (From Han and co-workers [175]). Human-induced pluripotent stem cells-derived cardiomyocytes were cultured on electrospun PCL fibers for 2 weeks. The fibers were coated with Matrigel® to enhance cell adhesion and cells were immunostained with DAPI and Phalloidin.

The scaffold topography plays a crucial role in inducing cardiomyocytes alignment which in turn influences contractile force [173]. To study the decrease of force contraction during the disease, cells have to be aligned to reproduce the *in vivo* conditions. Aligned cells are more mature and orientate toward a physiological behavior [107] because they have gap junctions and visible sarcomeres. Cardiomyocytes cultured *in vitro* on plastic are randomly aligned. As a consequence, the distribution of their contractile force is in all directions and so is not efficient [174]. To align cells, a scaffold needs to be anisotropic, *i.e.* to possess a preferential orientation. Intrinsic properties of the materials or shaping can create anisotropy in the scaffold and then cardiomyocytes will follow this anisotropy, *i.e.* they will be aligned (Fig. 6). We can notice several types of anisotropy: the first one is the anisotropy due to the shaping process such as electrospinning. Electrospinning parameters can be tuned to obtain aligned fibers. The second depends on the intrinsic properties of the material to form aligned fibers (extruded collagen, for instance). Last, cell anisotropy can be generated by a post processing step using mechanical stretching that forces the cells to align.

#### 4.4.1 Electrospinning

Using the electrostatic force, the electrospinning technique produces polymeric fibers ranging from nanoscale to microscale [176,177]. A highly intense electric field (1–10 kV) charges two electrodes with opposite polarities. The first electrode is immersed in the polymeric solution and the second electrode is placed on the collector. When the electrical field overcomes the resistance of the surface tension in the polymeric solution, the electrically charged solution is ejected towards the collector. The polymeric jet is stretched during this step and long and thin fibers are produced. By changing the rotating speed of the collector, aligned fibers or randomly organized fibers are obtained. This shaping process creates anisotropic material when fibers are aligned.

Electrospun Polyurethane fibers of around 2 μm in diameter can be used for cardiomyocyte culture after coating with fibronectin. This scaffold allows for cardiomyocyte alignment and synchronous contraction along the main axis of electrospunned fibers [178]. In addition, cells develop an anisotropic organization (i.e. aligned cells) with mature sarcomeres. Smaller fibers with polyvinylidene fluoride-trifluoroethylene (PVDF-TrFE) – 550 nm – [129] or PCL – 250 nm – in diameter [126] are obtained to create a fibrous substrate. Natural polymers are more difficult to process due to denaturation and are often inhomogeneous in diameter. However, polyglycerol sebacate fibers with a collagen I core were produced by electrospinning [46] as well as silk fibroin [176] or chitosan [179] fibers. Matthews and co-workers succeeded in optimizing collagen processes to obtain fibers of 250 nm in diameter with the structural organization of collagen fibrils – with the 67 nm D banding pattern –, a characteristic of native collagen [180]. Joanne and co-workers obtained larger fibers from 600 nm to 2 μm and they demonstrated the cytocompatibility of collagen electrospun subtrates [181]. IPS-CM survive on this substrate because the collagen and the high porosity improves cell colonization in the substrate.

The main limitation of such a process is the poor repeatability when natural polymers are used and only thin materials are obtained preventing organotypic organization.

#### 4.4.2 Intrinsic anisotropy of fibrillar biopolymers

Another approach to generate anisotropy is the use of an intrinsic anisotropic material. Many natural polymers such as collagen or fibrin form fibers. These fibers can be aligned within the biomaterial and the resulting scaffold is anisotropic.

The collagen I fibrillogenesis process generates fibrils of between 10 and 100 nm in diameter that can be aligned with a controlled extrusion process. Using a syringe and a well-adapted needle, the shearing conditions are adapted to form collagen threads with aligned fibers [182]. The needle diameter depends on the type of biopolymer used. Hyaluronic acid/collagen I bio ink forms aligned fibers with a 15G needle [183] whereas a 25G needle is required to align collagen I fibers within a decellularized ECM bioink [184]. This approach has also been used with Poly(lactic-co-glycolic acid) (PLGA) with a good fiber alignment. Cells seeded on this substrate follow the orientation of the substrate and align [185].

Fibrinogen mixed with thrombin self-assemble into fibers to form a hydrogel. By constraining this hydrogel, fibrin fibers align and the substrate becomes anisotropic [186]. Cardiomyocytes can be embedded into the fibrin hydrogel and they will survive and remodel their substrate thanks to the mesh structure [186].

As an alternative, a high magnetic field can be used to orientate diamagnetic molecules. Collagen I is a diamagnetic molecule due to its high aspect ratio and its intrinsic properties [187,188]. The magnetic field induces the rotation of collagen molecules to align in one direction during the self-assembly process [189,190]. However, this approach requires a very strong magnetic field and few structures are available to produce such conditions.

#### 4.4.3 Induced anisotropy by mechanical stretching

This strategy uses mechanical forces to induce cell alignment once the cells are cultured within the scaffold. Before the stimulation, the cells are randomly dispersed but after cycles of stretching they align along the stretching axis. Mechanical stress conditioning along one axis promotes a 2-fold increase in cardiomyocyte size and cell alignment [139]. The external stimulation is not always required because a fibrin hydrogel fixed between two PDMS pillars will contract along the axis of attachment. The PDMS pillars bend during the contraction and the elongation induces alignment of cells [90]. This is the most widely used technique to align cardiomyocytes encapsulated within a hydrogel to form a microtissue mimicking DCM [191].

ECM anisotropy is a crucial parameter to consider for mimicking the topography of a physiological heart. Electrospinning of fibers does not seem to be relevant due to the scaffold thinness and the use of synthetic polymers. Hence, the use of a natural polymer with intrinsic anisotropic fibers seems suitable for 3D cardiac modelling which can be aligned when the polymeric solution is extruded while 3D printing. As a result, this technique allows for the generation of macroporous and anisotropic scaffolds.

## 5 Physiological evaluation of a 3D cardiac model

Among the read-outs, contractility seems to be the most relevant parameter with which to study DCM. The tissue displacement during contraction and its frequency give an estimation of contractility and beat rate. Force measurements on isolated cardiomyofibrils show a reduced tension in sarcomeres of around 25% in DCM cells. Combined with the reduced population and the forming fibrosis, these consequences drastically reduce the force contraction in the patient's heart [192].

At the cellular level, immunostaining image analysis gives information on parameters such as the sarcomere size during contraction and stretching. Images are modified with a 2D Fourier transform to extract sarcomere content and cell length. The mean length between sarcomeres and the aspect ratio of cardiomyocytes indicates their ability to beat [56]. A new software proposes to analyze time lapse of beating cardiomyocytes and to extract PDMS post deflection, fractional shortening of sarcomere length and beating rate thanks to an algorithm [193]. Human involvement is limited and analysis of the images and video is by algorithm. New techniques using live imaging allow a spatiotemporal analysis. For instance, voltage-sensitive dies enable the monitoring of the action potential propagation in a cell sheet or a 3D culture [194]. Electrodes placed on the culture surface report the electric potential and its propagation wave when cardiomyocytes are aligned. These spatiotemporal methods afford a better understanding of the cell behavior of cardiomyocytes collected from healthy or sick patients [195]. Yang and Hsu developed a piezo electric membrane to record spontaneous contraction of aligned cells in 2D^96^. To convert this technique into 3D measurements, Tian and co-workers elaborated a highly porous electronic mesh integrated in a PLGA electrospun fiber scaffold. After seeding, cardiomyocytes interact with the electronic mesh that will record the electric propagation in a 3D environment. This electronic mesh does not affect tissue formation and growth because of its flexibility and porosity. This set up allows monitoring of extracellular potential at different locations with a high spatiotemporal resolution [196]. This approach is a major research orientation to design bionic tissues with built-in electronics to monitor cell function and to provide electrical stimulation to cardiomyocytes [197]. To go further, optogenetics tools have been adapted to cardiomyocytes studies [198]. Light sensitive channels have been transfected to cardiomyocytes owing to lentivirus. With these channels, cells are sensitive to a specific wave length and their contraction can be triggered and controlled [199]. Moreover, Park and co-workers developed a set up able to combine optical mapping and optogenetic stimulation. Because of the light sensitivity they were able to change the shapes of the action potential while recording them. This technique allows a very high spatial and temporal resolution. The electrical signal was recording with a voltage sensitive dye [200] (see Table 3).

**Table 3:** Comparison of the different strategies to assess the performance of a cardiac model.

Techniques	Video	AFM/Cantilever	PDMS pillars	Force transducer	Electroresponsive substrate	Fluorescent microbeads	Optogenetic
Reads-out	Beating frequency	Beating frequency	Beating frequency	Beating frequency	Beating frequency	Beating frequency	Beating frequency
		Vertical force	Tissue contraction	Tissue contraction		Cell displacement	Electrical wave
		Cellular stiffness					

Cell features	Cell length/width ratio during contraction	Contractile amplitude & force	Average cell length/width ratio during contraction Contractile amplitude & force	Average cell length/width ratio during contraction Contractile amplitude & force	Electrical currents inside the cells	Cell length/width ratio during contraction	Action potential propagation

Type of models	2D and 3D	2D and 3D	3D	3D or single cell	2D and 3D	2D and 3D	2D and 3D

Advantages	Easy to handle	Very accurate	Non invasive	Easy to handle	Live imaging	Live imaging	High temporal and spatial resolution
	Non invasive		Easy to handle		Visible electric wave of contraction	Non invasive	
	Live imaging						

Limitations	No force measurement	Long time acquisition	Not accurate, only a global force	Global force contraction or single cell	Substrate interfere with cells	More accurate with single cells	Require to modify cells
		Local acquisition	Indirect measurement				

References	[56,194]	[203,205]	[9,56,193]	[47,202]	[96,196]	[204]	[[198], [199], [200]]

**Fig. 7:**
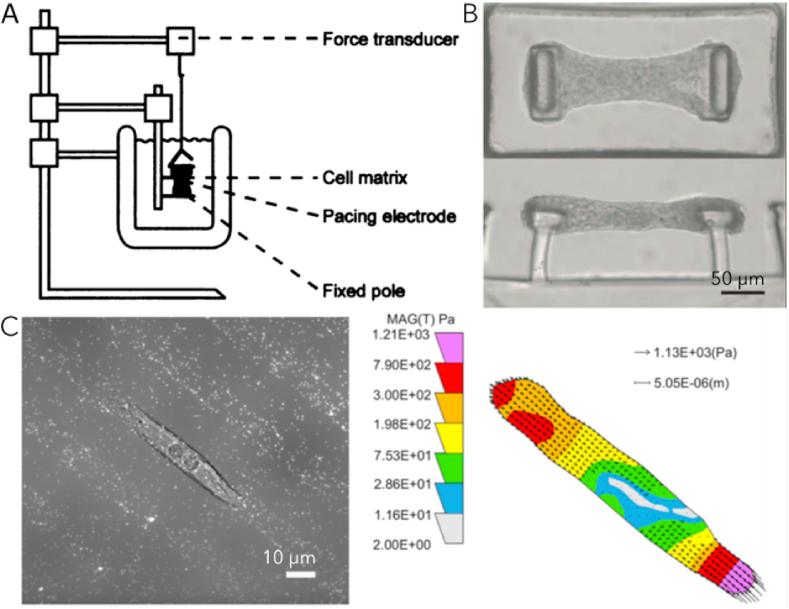
**Different strategies to measure cardiomyocyte contractility.** A- Using a force transducer and a fixed pole, the contractility of the whole gel is measured [47]. B- The mix of cell and hydrogel is molded between two PDMS pillars. By following the displacement of the pillars, the force contraction of the gel is measured [56].C- Fluorescent beads are mixed with the hydrogel. During cultivation, bead movements are recorded and allow for contraction measurement of the cell [204]. The heat map represents the traction force applied by the cell on the substrate (1 Pa = 10^−3^ N mm^−2^).

In most cases, the force contraction of an engineered tissue is directly measured with a force transducer [47,87,146] (Fig. 7A) or indirectly by the measurement of displacement of PDMS pillars [9,201] (Fig. 7B). In the first case, the gel is attached to a fixed part and to a force transducer that will measure the force contraction. In the second case, the gel grows between two PDMS pillars and its contraction will induce pillar deflection that is recorded. Using these systems, cardiomyocytes have a lower beating rate and a reduced force contraction than the physiological heart. This difference may be due to a lack of cell alignment and a lower cell density that induce a poor conduction. Force transducers can be miniaturized to study single cell contraction with very high accuracy [202]. Combined with a video microscope, this technique allows observation of cell striation and contraction of cardiac myocytes. However, some examples demonstrate that a single cell assay was not relevant to show difference in contractility between cells affected by DCM (titin mutation) and healthy cells [56]. Moving to 3D tissue models with cells randomly cultured into a hydrogel, these showed that the mutated line generates only 50% of the contractile force compared to the control line. This study proves the lower ability to beat of mutated cardiomyocytes. For this purpose, fluorescent microbeads were mixed with PDMS to create pillars on top of which the engineered heart tissue is anchored. With fluorescent imaging and tracking of the beads, cell contractility is assessed within the entire tissue [56]. Other techniques were developed based on the displacement of a cantilever brought into contact with cardiac myocytes [203] or using atomic force microscopy but these techniques are more suited to 2D cultures. The different techniques are compared in Table 3.

## 6 Future perspectives

Most researchers in the field of cardiac tissue engineering must seek to combine a suitable biomaterial with iPS-CM to create a reliable cardiac model. This approach is particularly relevant with DCM since this disease affects not only the cells but also the cardiac ECM. By optimizing the bioprinting techniques and the macroporosity generation, we should soon be able to create a porous substrate allowing for iPS-CM cultivation. Mimicking the stiffness of DCM extracellular matrix is the simplest parameter to reproduce *in vitro*. The porosity is another crucial point to focus on since the vascularization network is reduced in DCM. Designing several biomaterials with distinct stiffness and porosity before adding iPS-CM could help to understand the impact of ECM changes on the evolution of contractile function impairment.

This iPS cell strategy is particularly well adapted to genetic disease modelling. Once the 3D cellularized scaffold is mature enough, the *in vitro* heart tissue will exhibit the disease features at the cell and tissue level [56,206]. Thanks to the CRISPR-Cas9 technology, it is now feasible to generate an artificial control of DCM cells. Both cellularized models will have the same genetic background corresponding to that of a specific patient. For a personalized medicine strategy, these models could be useful for drug testing to find the appropriate treatment for a specific patient. The biomaterial contractility and the cellular morphology could be relevant read-outs. The optical mapping technique with a voltage sensitive dye seems to be the most promising strategy to observe the signal conduction between cells [62,95] and evaluate the treatment efficacy. The video analysis and comparison with the control will reveal any improvements in the treated diseased model.

## 7 Conclusion

The development of a fully functional cardiac tissue model of DCM could not be completed without a close collaboration between material physico-chemistry and cell biology. With the development of iPS cells, it is now possible to derive pathological cells from blood circulating cells. To reproduce the cellular diversity of heart tissue, other cells such as fibroblasts and endothelial cells can be co-cultivated with iPS-CM with the aim of recreating the physiological cell interactions. These advances in cardiac cell biology must be combined with a suitable 3D scaffold mimicking the heart ECM to set-up adequate cell/ECM interactions. Advances in biomaterial design techniques such as 3D printing enable fabrication of an artificial cardiac ECM with adequate stiffness, composition and shape. Macroporosity and anisotropy are also crucial to reproduce the cell microenvironment in a healthy or a diseased heart. This porosity allows the cell organotypic organization and the development of a primitive vascular network when endothelial cells are added. This vascular network can be perfused to reproduce the blood pressure inside the heart tissue. Furthermore, biomaterials nowadays allow greater innovation through the addition of drug release particles or electronical mesh to produce and record cell contraction. By combining all these technologies, it appears possible to create a functional cardiac tissue model of genetic DCM.

## Funding

This research was partly supported by 10.13039/100012946Sorbonne Université, 10.13039/501100004794CNRS, 10.13039/501100001677INSERM. Christophe Hélary is supported by the 10.13039/100013465AFM-Téléthon (contract number: 22142). Onnik Agbulut and Pierre Joanne are supported by the 10.13039/100013465AFM-Téléthon (contract numbers: 21833 and 22142), the 10.13039/501100003100Fédération Française de Cardiologie and by the 10.13039/501100017007Ile-de-France Region in the framework of Respore, the Île-de-France network of Excellence in Porous Solids. Marie Camman is supported by a Ph.D. fellowship from 10.13039/100012946Sorbonne Université.

## Declaration of interests

The authors declare no conflict of interests relevant to this work.

## Declaration of competing interest

The authors declare that they have no known competing financial interests or personal relationships that could have appeared to influence the work reported in this paper.

## References

[1] Ponikowski P., Voors A.A., Anker S.D., Bueno H., Cleland J.G.F., Coats A.J.S., Falk V., González-Juanatey J.R., Harjola V.-P., Jankowska E.A., Jessup M., Lindeai C., Nihoyannopoulos P., Parissis J.T., Pieske B., Riley J.P., Rosano G.M.C., Ruilope L.M., Ruschitzka F., Rutten F.H., van der Meer P., Authors/Task Force Members, Document Reviewers (2016). ESC guidelines for the diagnosis and treatment of acute and chronic heart failure: the task force for the diagnosis and treatment of acute and chronic heart failure of the European society of cardiology (ESC). Developed with the spec. Eur. J. Heart Fail..

[2] Virani S.S., Alonso A., Benjamin E.J., Bittencourt M.S., Callaway C.W., Carson A.P., Chamberlain A.M., Chang A.R., Cheng S., Delling F.N., Djousse L., Elkind M.S.V., Ferguson J.F., Fornage M., Khan S.S., Kissela B.M., Knutson K.L., Kwan T.W., Lackland D.T., Lewis T.T., Lichtman J.H., Longenecker C.T., Loop M.S., Lutsey P.L., Martin S.S., Matsushita K., Moran A.E., Mussolino M.E., Perak A.M., Rosamond W.D., Roth G.A., Sampson U.K.A., Satou G.M., Schroeder E.B., Shah S.H., Shay C.M., Spartano N.L., Stokes A., Tirschwell D.L., VanWagner L.B., Tsao C.W. (2020). On behalf of the American heart association council on epidemiology and prevention statistics committee and stroke statistics subcommittee. Heart disease and stroke statistics—2020 update: a report from the American heart association. Circulation.

[3] Seferović P.M., Polovina M., Bauersachs J., Arad M., Gal T.B., Lund L.H., Felix S.B., Arbustini E., Caforio A.L.P., Farmakis D., Filippatos G.S., Gialafos E., Kanjuh V., Krljanac G., Limongelli G., Linhart A., Lyon A.R., Maksimović R., Miličić D., Milinković I., Noutsias M., Oto A., Oto Ö., Pavlović S.U., Piepoli M.F., Ristić A.D., Rosano G.M.C., Seggewiss H., Ašanin M., Seferović J.P., Ruschitzka F., Čelutkiene J., Jaarsma T., Mueller C., Moura B., Hill L., Volterrani M., Lopatin Y., Metra M., Backs J., Mullens W., Chioncel O., Boer R.A., Anker S., Rapezzi C., Coats A.J.S., Tschöpe C. (2019). Heart failure in cardiomyopathies: a position paper from the heart failure association of the European society of cardiology. Eur. J. Heart Fail..

[4] Pinto Y.M., Elliott P.M., Arbustini E., Adler Y., Anastasakis A., Böhm M., Duboc D., Gimeno J., de Groote P., Imazio M., Heymans S., Klingel K., Komajda M., Limongelli G., Linhart A., Mogensen J., Moon J., Pieper P.G., Seferovic P.M., Schueler S., Zamorano J.L., Caforio A.L.P., Charron P. (2016). Proposal for a revised definition of dilated cardiomyopathy, hypokinetic non-dilated cardiomyopathy, and its implications for clinical practice: a position statement of the ESC working group on myocardial and pericardial diseases. Eur. Heart J..

[5] Peters S., Johnson R., Birch S., Zentner D., Hershberger R.E., Fatkin D. (2020). Familial dilated cardiomyopathy. Heart Lung Circ..

[6] McNally E.M., Mestroni L. (2017). Dilated cardiomyopathy: genetic determinants and mechanisms. Circ. Res..

[7] Parlakian A., Charvet C., Escoubet B., Mericskay M., Molkentin J.D., Gary-Bobo G., De Windt L.J., Ludosky M.-A., Paulin D., Daegelen D., Tuil D., Li Z. (2005). Temporally controlled onset of dilated cardiomyopathy through disruption of the *SRF* gene in adult heart. Circulation.

[8] Takahashi K., Yamanaka S. (2006). Induction of pluripotent stem cells from mouse embryonic and adult fibroblast cultures by defined factors. Cell.

[9] Boudou T., Legant W.R., Mu A., Borochin M.A., Thavandiran N., Radisic M., Zandstra P.W., Epstein J.A., Margulies K.B., Chen C.S. (2012).

[10] Streckfuss-Bömeke K., Tiburcy M., Fomin A., Luo X., Li W., Fischer C., Özcelik C., Perrot A., Sossalla S., Haas J., Vidal R.O., Rebs S., Khadjeh S., Meder B., Bonn S., Linke W.A., Zimmermann W.-H., Hasenfuss G., Guan K. (2017). Severe DCM phenotype of patient harboring RBM20 mutation S635A can Be modeled by patient-specific induced pluripotent stem cell-derived cardiomyocytes. J. Mol. Cell. Cardiol..

[11] Hirota Y., Shimizu G., Kaku K., Saito T., Kino M., Kawamura K. (1984). Mechanisms of compensation and decompensation in dilated cardiomyopathy. Am. J. Cardiol..

[12] Narayanan K., Reinier K., Teodorescu C., Uy‐Evanado A., Aleong R., Chugh H., Nichols G.A., Gunson K., London B., Jui J., Chugh S.S. (2014). Left ventricular diameter and risk stratification for sudden cardiac death. J. Am. Heart Assoc..

[13] Yu Y., Yu S., Tang X., Ren H., Li S., Zou Q., Xiong F., Zheng T., Gong L. (2017). Evaluation of left ventricular strain in patients with dilated cardiomyopathy. J. Int. Med. Res..

[14] Frangogiannis N.G. (2017). The extracellular matrix in myocardial injury, repair, and remodeling. J. Clin. Invest..

[15] Mihl C., Dassen W.R.M., Kuipers H. (2008). Cardiac remodelling: concentric versus eccentric hypertrophy in strength and endurance athletes. Neth. Heart J..

[16] Jefferies J.L., Towbin J.A. (2010). Dilated cardiomyopathy. Lancet.

[17] Saini H., Tabtabai S., Stone J.R., Ellinor P.T. (2014). Pathophysiology of cardiomyopathies. Cellular and Molecular Pathobiology of Cardiovascular Disease.

[18] Lynch T.L., Ismahil M.A., Jegga A.G., Zilliox M.J., Troidl C., Prabhu S.D., Sadayappan S. (2017). Cardiac inflammation in genetic dilated cardiomyopathy caused by MYBPC3 mutation. J. Mol. Cell. Cardiol..

[19] Bergmann O., Zdunek S., Felker A., Salehpour M., Alkass K., Bernard S., Sjostrom S.L., Szewczykowska M., Jackowska T., dos Remedios C., Malm T., Andrä M., Jashari R., Nyengaard J.R., Possnert G., Jovinge S., Druid H., Frisén J. (2015). Dynamics of cell generation and turnover in the human heart. Cell.

[20] Huang C.Y., Peres Moreno Maia-Joca R., Ong C.S., Wilson I., DiSilvestre D., Tomaselli G.F., Reich D.H. (2020). Enhancement of human IPSC-derived cardiomyocyte maturation by chemical conditioning in a 3D environment. J. Mol. Cell. Cardiol..

[21] Roura S. (2009).

[22] Roura S., Planas F., Prat-Vidal C., Leta R., Soler-Botija C., Carreras F., Llach A., Hove-Madsen L., Lladó G.P., Farré J., Cinca J., Bayes-Genis A. (2007). Idiopathic dilated cardiomyopathy exhibits defective vascularization and vessel formation. Eur. J. Heart Fail..

[23] Vikhorev P., Vikhoreva N. (2018). Cardiomyopathies and related changes in contractility of human heart muscle. Int. J. Mol. Sci..

[24] Gaetani R., Zizzi E.A., Deriu M.A., Morbiducci U., Pesce M., Messina E. (2020). When stiffness matters: mechanosensing in heart development and disease. Front. Cell Dev. Biol..

[25] Vikhorev P.G., Smoktunowicz N., Munster A.B., Copeland O., Kostin S., Montgiraud C., Messer A.E., Toliat M.R., Li A., dos Remedios C.G., Lal S., Blair C.A., Campbell K.S., Guglin M., Richter M., Knöll R., Marston S.B. (2017). Abnormal contractility in human heart myofibrils from patients with dilated cardiomyopathy due to mutations in TTN and contractile protein genes. Sci. Rep..

[26] Ribeiro M.C., Slaats R.H., Schwach V., Rivera-Arbelaez J.M., Tertoolen L.G.J., van Meer B.J., Molenaar R., Mummery C.L., Claessens M.M.A.E., Passier R. (2020). A cardiomyocyte show of force: a fluorescent alpha-actinin reporter line sheds light on human cardiomyocyte contractility versus substrate stiffness. J. Mol. Cell. Cardiol..

[27] Dorn G.W., Vega R.B., Kelly D.P. (2015). Mitochondrial biogenesis and dynamics in the developing and diseased heart. Genes Dev..

[28] Ritterhoff J., Tian R. (2017). Metabolism in cardiomyopathy: every substrate matters. Cardiovasc. Res..

[29] Gosselin-Badaroudine P., Keller D.I., Huang H., Pouliot V., Chatelier A., Osswald S., Brink M., Chahine M. (2012). A proton leak current through the cardiac sodium channel is linked to mixed arrhythmia and the dilated cardiomyopathy phenotype. PloS One.

[30] Voorhees A.P., Han H.-C., Terjung R. (2015). Biomechanics of cardiac function. Comprehensive Physiology.

[31] Domian I.J., Yu H., Mittal N. (2017). On materials for cardiac tissue engineering. Adv. Healthc. Mater..

[32] Cojan-Minzat B.O., Zlibut A., Agoston-Coldea L. (2020). Non-ischemic dilated cardiomyopathy and cardiac fibrosis. Heart Fail. Rev..

[33] Louzao-Martinez L., Vink A., Harakalova M., Asselbergs F.W., Verhaar M.C., Cheng C. (2016). Characteristic adaptations of the extracellular matrix in dilated cardiomyopathy. Int. J. Cardiol..

[34] Pankuweit S., Ruppert V., Maisch B. (2004). Inflammation in dilated cardiomyopathy. Herz.

[35] Kapelko V.I. (2001). Extracellular matrix alterations in cardiomyopathy: the possible crucial role in the dilative form. Europace.

[36] Beltrami C., Finato N., Rocco M., Feruglio G., Puricelli C., Cigola E., Sonnenblick E., Olivetti G., Anversa P. (1995). The cellular basis of dilated cardiomyopathy in humans. J. Mol. Cell. Cardiol..

[37] Stoker M.E., Gerdes A.M., May J.F. (1982). Regional differences in capillary density and myocyte size in the normal human heart. Anat. Rec..

[38] Figulla H.R., Vetterlein F., Wiegand V., Schüler S., Kreuzer H., Kaltenbach M., Hopf R., Kunkel B. (1988). Capillary density and oxygen supply in human dilated cardiomyopathy. New Aspects of Hypertrophic Cardiomyopathy.

[39] Makarenko I., Opitz C.A., Leake M.C., Neagoe C., Kulke M., Gwathmey J.K., del Monte F., Hajjar R.J., Linke W.A. (2004). Passive stiffness changes caused by upregulation of compliant titin isoforms in human dilated cardiomyopathy hearts. Circ. Res..

[40] Andrew R. (2003). Marks. Calcium and the Heart: A Question of Life and Death.

[41] Razeghi P., Young M.E., Alcorn J.L., Moravec C.S., Frazier O.H., Taegtmeyer H. (2001). Metabolic gene expression in fetal and failing human heart. Circulation.

[42] Dávila-Román V.G., Vedala G., Herrero P., de las Fuentes L., Rogers J.G., Kelly D.P., Gropler R.J. (2002). Altered myocardial fatty acid and glucose metabolism in idiopathic dilated cardiomyopathy. J. Am. Coll. Cardiol..

[43] Groenewegen W.A., Firouzi M., Bezzina C.R., Vliex S., van Langen I.M., Sandkuijl L., Smits J.P.P., Hulsbeek M., Rook M.B., Jongsma H.J., Wilde A.A.M. (2003). A cardiac sodium channel mutation cosegregates with a rare Connexin40 genotype in familial atrial standstill. Circ. Res..

[44] Dar A., Shachar M., Leor J., Cohen S. (2002). Optimization of cardiac cell seeding and distribution in 3D porous alginate scaffolds. Biotechnol. Bioeng..

[45] Blan N.R., Birla R.K. (2008). Design and fabrication of heart muscle using scaffold-based tissue engineering. J. Biomed. Mater. Res..

[46] Ravichandran R. (2013). Cardiogenic differentiation of mesenchymal stem cells on elastomeric poly (glycerol sebacate)/collagen core/shell fibers. World J. Cardiol..

[47] Eschenhagen T., Fink C., Remmers U., Scholz H., Wattchow J., Weil J., Zimmermann W., Dohmen H.H., Schäfer H., Bishopric N., Wakatsuki T., Elson E.L. (1997). Three‐dimensional reconstitution of embryonic cardiomyocytes in a collagen matrix: a new heart muscle model system. Faseb. J..

[48] Davidson M., Nesti C., Palenzuela L., Walker W., Hernandez E., Protas L., Hirano M., Isaac N. (2005). Novel cell lines derived from adult human ventricular cardiomyocytes. J. Mol. Cell. Cardiol..

[49] Watkins S.J., Borthwick G.M., Arthur H.M. (2011). The H9C2 cell line and primary neonatal cardiomyocyte cells show similar hypertrophic responses in vitro. *Vitro cell*. Dev. Biol. Anim..

[50] Burridge P.W., Matsa E., Shukla P., Lin Z.C., Churko J.M., Ebert A.D., Lan F., Diecke S., Huber B., Mordwinkin N.M., Plews J.R., Abilez O.J., Cui B., Gold J.D., Wu J.C. (2014). Chemically defined generation of human cardiomyocytes. Nat. Methods.

[51] Engler A.J., Sen S., Sweeney H.L., Discher D.E. (2006). Matrix elasticity directs stem cell lineage specification. Cell.

[52] Young J.L., Kretchmer K., Ondeck M.G., Zambon A.C., Engler A.J. (2015). Mechanosensitive kinases regulate stiffness-induced cardiomyocyte maturation. Sci. Rep..

[53] Chu A.J., Zhao E.J., Chiao M., Lim C.J. (2020). Co-culture of induced pluripotent stem cells with cardiomyocytes is sufficient to promote their differentiation into cardiomyocytes. PloS One.

[54] Kobold S., Guhr A., Kurtz A., Löser P. (2015). Human embryonic and induced pluripotent stem cell research trends: complementation and diversification of the field. Stem Cell Rep..

[55] Mallon B.S., Hamilton R.S., Kozhich O.A., Johnson K.R., Fann Y.C., Rao M.S., Robey P.G. (2014). Comparison of the molecular profiles of human embryonic and induced pluripotent stem cells of isogenic origin. Stem Cell Res..

[56] Hinson J.T., Chopra A., Nafissi N., Polacheck W.J., Benson C.C., Swist S., Gorham J., Yang L., Schafer S., Sheng C.C., Haghighi A., Homsy J., Hubner N., Church G., Cook S.A., Linke W.A., Chen C.S., Seidman J.G., Seidman C.E. (2015). Titin mutations in IPS cells define sarcomere insufficiency as a cause of dilated cardiomyopathy. Science.

[57] Siu C.-W., Lee Y.-K., Ho J.C.-Y., Lai W.-H., Chan Y.-C., Ng K.-M., Wong L.-Y., Au K.-W., Lau Y.-M., Zhang J., Lay K.W., Colman A., Tse H.-F. (2012). Modeling of lamin A/C mutation premature cardiac aging using patient-specific induced pluripotent stem cells. Aging.

[58] Musunuru K., Sheikh F., Gupta R.M., Houser S.R., Maher K.O., Milan D.J., Terzic A., Wu J.C. (2018). Induced pluripotent stem cells for cardiovascular disease modeling and precision medicine: a scientific statement from the American heart association. Circ. Genom. Precis. Med..

[59] van Mil A., Balk G.M., Neef K., Buikema J.W., Asselbergs F.W., Wu S.M., Doevendans P.A., Sluijter J.P.G. (2018). Modelling inherited cardiac disease using human induced pluripotent stem cell-derived cardiomyocytes: progress, pitfalls, and potential. Cardiovasc. Res..

[60] Long C., Li H., Tiburcy M., Rodriguez-Caycedo C., Kyrychenko V., Zhou H., Zhang Y., Min Y.-L., Shelton J.M., Mammen P.P.A., Liaw N.Y., Zimmermann W.-H., Bassel-Duby R., Schneider J.W., Olson E.N. (2018). Correction of diverse muscular dystrophy mutations in human engineered heart muscle by single-site genome editing. Sci. Adv..

[61] Rebs S., Sedaghat-Hamedani F., Kayvanpour E., Meder B., Streckfuss-Bömeke K. (2020). Generation of pluripotent stem cell lines and CRISPR/Cas9 modified isogenic controls from a patient with dilated cardiomyopathy harboring a RBM20 p.R634W mutation. Stem Cell Res..

[62] Kupfer M.E., Lin W.-H., Ravikumar V., Qiu K., Wang L., Gao L., Bhuiyan D., Lenz M., Ai J., Mahutga R.R., Townsend D., Zhang J., McAlpine M.C., Tolkacheva E.G., Ogle B.M. (2020). *In situ* expansion, differentiation and electromechanical coupling of human cardiac muscle in a 3D bioprinted. Chambered Organoid. *Circ. Res.*.

[63] Buikema J.W., Lee S., Goodyer W.R., Maas R.G., Chirikian O., Li G., Miao Y., Paige S.L., Lee D., Wu H., Paik D.T., Rhee S., Tian L., Galdos F.X., Puluca N., Beyersdorf B., Hu J., Beck A., Venkamatran S., Swami S., Wijnker P., Schuldt M., Dorsch L.M., van Mil A., Red-Horse K., Wu J.Y., Geisen C., Hesse M., Serpooshan V., Jovinge S., Fleischmann B.K., Doevendans P.A., van der Velden J., Garcia K.C., Wu J.C., Sluijter J.P.G., Wu S.M. (2020). Wnt activation and reduced cell-cell contact synergistically induce massive expansion of functional human IPSC-derived cardiomyocytes. Cell Stem Cell.

[64] Huebsch N., Loskill P., Deveshwar N., Spencer C.I., Judge L.M., Mandegar M.A., Fox B., Mohamed T.M.A., Ma Z., Mathur A., Sheehan A.M., Truong A., Saxton M., Yoo J., Srivastava D., Desai T.A., So P.-L., Healy K.E., Conklin B.R. (2016). Miniaturized IPS-Cell-Derived cardiac muscles for physiologically relevant drug response analyses. Sci. Rep..

[65] Feric N.T., Radisic M. (2016). Maturing human pluripotent stem cell-derived cardiomyocytes in human engineered cardiac tissues. Adv. Drug Deliv. Rev..

[66] Ge F., Wang Z., Xi J.J. (2019). Engineered maturation approaches of human pluripotent stem cell-derived ventricular cardiomyocytes. Cells.

[67] Jiang Y., Park P., Hong S.-M., Ban K. (2018). Maturation of cardiomyocytes derived from human pluripotent stem cells: current strategies and limitations. Mol. Cell.

[68] Lundy S.D., Zhu W.-Z., Regnier M., Laflamme M.A. (2013). Structural and functional maturation of cardiomyocytes derived from human pluripotent stem cells. Stem Cell. Dev..

[69] Chung J., Shum-Tim D. (2012). Neovascularization in tissue engineering. Cells.

[70] Gay M.S., Dasgupta C., Li Y., Kanna A., Zhang L. (2016). Dexamethasone induces cardiomyocyte terminal differentiation via epigenetic repression of cyclin D2 gene. J. Pharmacol. Exp. Therapeut..

[71] Falcón D., González-Montelongo R., Sánchez de Rojas-de Pedro E., Ordóñez A., Ureña J., Castellano A. (2018). Dexamethasone-induced upregulation of CaV3.2 T-type Ca2+ channels in rat cardiac myocytes. J. Steroid Biochem. Mol. Biol..

[72] Yang X., Rodriguez M., Pabon L., Fischer K.A., Reinecke H., Regnier M., Sniadecki N.J., Ruohola-Baker H., Murry C.E. (2014). Tri-iodo-L-thyronine promotes the maturation of human cardiomyocytes-derived from induced pluripotent stem cells. J. Mol. Cell. Cardiol..

[73] Costa A., Rossi E., Scicchitano B.M., Coletti D., Moresi V., Adamo S. (2014). Neurohypophyseal hormones: novel actors of striated muscle development and homeostasis. Eur. J. Transl. Myol..

[74] Gutkowska J., Jankowski M. (2012). Oxytocin revisited: its role in cardiovascular regulation. J. Neuroendocrinol..

[75] Paquin J., Danalache B.A., Jankowski M., McCann S.M., Gutkowska J. (2002). Oxytocin induces differentiation of P19 embryonic stem cells to cardiomyocytes. Proc. Natl. Acad. Sci. Unit. States Am..

[76] Horikoshi Y., Yan Y., Terashvili M., Wells C., Horikoshi H., Fujita S., Bosnjak Z., Bai X. (2019). Fatty acid-treated induced pluripotent stem cell-derived human cardiomyocytes exhibit adult cardiomyocyte-like energy metabolism phenotypes. Cells.

[77] Correia C., Serra M., Espinha N., Sousa M., Brito C., Burkert K., Zheng Y., Hescheler J., Carrondo M.J.T., Šarić T., Alves P.M. (2014). Combining hypoxia and bioreactor hydrodynamics boosts induced pluripotent stem cell differentiation towards cardiomyocytes. Stem Cell Rev. Rep..

[78] Fleischer S., Jahnke H.-G., Fritsche E., Girard M., Robitzki A.A. (2019). Comprehensive human stem cell differentiation in a 2D and 3D mode to cardiomyocytes for long-term cultivation and multiparametric monitoring on a multimodal microelectrode array setup. Biosens. Bioelectron..

[79] Magrofuoco E., Flaibani M., Giomo M., Elvassore N. (2019). Cell culture distribution in a three-dimensional porous scaffold in perfusion bioreactor. Biochem. Eng. J..

[80] Pagliari S., Tirella A., Ahluwalia A., Duim S., Goumans M.-J., Aoyagi T., Forte G. (2014). A multistep procedure to prepare pre-vascularized cardiac tissue constructs using adult stem sells, dynamic cell cultures, and porous scaffolds. Front. Physiol..

[81] Madden L.R., Mortisen D.J., Sussman E.M., Dupras S.K., Fugate J.A., Cuy J.L., Hauch K.D., Laflamme M.A., Murry C.E., Ratner B.D. (2010). Proangiogenic scaffolds as functional templates for cardiac tissue engineering. Proc. Natl. Acad. Sci. Unit. States Am..

[82] Kolesky D.B., Homan K.A., Skylar-Scott M.A., Lewis J.A. (2016). Three-Dimensional bioprinting of thick vascularized tissues. Proc. Natl. Acad. Sci. Unit. States Am..

[83] Sadahiro T. (2019). Cardiac regeneration with pluripotent stem cell-derived cardiomyocytes and direct cardiac reprogramming. Regen. Ther..

[84] Kolanowski T.J., Busek M., Schubert M., Dmitrieva A., Binnewerg B., Pöche J., Fisher K., Schmieder F., Grünzner S., Hansen S., Richter A., El-Armouche A., Sonntag F., Guan K. (2020). Enhanced structural maturation of human induced pluripotent stem cell-derived cardiomyocytes under a controlled microenvironment in a microfluidic system. Acta Biomater..

[85] Kaiser N.J., Coulombe K.L.K. (2015). Physiologically inspired cardiac scaffolds for tailored *in vivo* function and heart regeneration. Biomed. Mater..

[86] Skylar-Scott M.A., Uzel S.G.M., Nam L.L., Ahrens J.H., Truby R.L., Damaraju S., Lewis J.A. (2019). Biomanufacturing of organ-specific tissues with high cellular density and embedded vascular channels. Sci. Adv..

[87] Zimmermann W.-H., Schneiderbanger K., Schubert P., Didié M., Münzel F., Heubach J.F., Kostin S., Neuhuber W.L., Eschenhagen T. (2002). Tissue engineering of a differentiated cardiac muscle construct. Circ. Res..

[88] Mihic A., Li J., Miyagi Y., Gagliardi M., Li S.-H., Zu J., Weisel R.D., Keller G., Li R.-K. (2014). The effect of cyclic stretch on maturation and 3D tissue formation of human embryonic stem cell-derived cardiomyocytes. Biomaterials.

[89] Tandon N., Cannizzaro C., Chao P.-H.G., Maidhof R., Marsano A., Au H.T.H., Radisic M., Vunjak-Novakovic G. (2009). Electrical stimulation systems for cardiac tissue engineering. Nat. Protoc..

[90] Ronaldson-Bouchard K., Ma S.P., Yeager K., Chen T., Song L., Sirabella D., Morikawa K., Teles D., Yazawa M., Vunjak-Novakovic G. (2018). Advanced maturation of human cardiac tissue grown from pluripotent stem cells. Nature.

[91] Sun X., Nunes S.S. (2017). Bioengineering approaches to mature human pluripotent stem cell-derived cardiomyocytes. Front. Cell Dev. Biol..

[92] Ahmed R.E., Anzai T., Chanthra N., Uosaki H. (2020). A brief review of current maturation methods for human induced pluripotent stem cells-derived cardiomyocytes. Front. Cell Dev. Biol..

[93] Sun N., Yazawa M., Liu J., Han L., Sanchez-Freire V., Abilez O.J., Navarrete E.G., Hu S., Wang L., Lee A., Pavlovic A., Lin S., Chen R., Hajjar R.J., Snyder M.P., Dolmetsch R.E., Butte M.J., Ashley E.A., Longaker M.T., Robbins R.C., Wu J.C. (2012). Patient-specific induced pluripotent stem cells as a model for familial dilated cardiomyopathy. Sci. Transl. Med..

[94] Shah D., Virtanen L., Prajapati C., Kiamehr M., Gullmets J., West G., Kreutzer J., Pekkanen-Mattila M., Heliö T., Kallio P., Taimen P., Aalto-Setälä K. (2019). Modeling of LMNA-related dilated cardiomyopathy using human induced pluripotent stem cells. Cells.

[95] Thompson S.A., Copeland C.R., Reich D.H., Tung L. (2011). Mechanical coupling between myofibroblasts and cardiomyocytes slows electric conduction in fibrotic cell monolayers. Circulation.

[96] Yang C.-F., Hsu Y.-H. (2019). Study of contraction profile of cardiomyocytes by using a piezoelectric membrane. 2019 41st Annual International Conference of the IEEE Engineering in Medicine and Biology Society (EMBC).

[97] Wang P.-Y., Yu J., Lin J.-H., Tsai W.-B. (2011). Modulation of alignment, elongation and contraction of cardiomyocytes through a combination of nanotopography and rigidity of substrates. Acta Biomater..

[98] Aamodt J.M., Grainger D.W. (2016). Extracellular matrix-based biomaterial scaffolds and the host response. Biomaterials.

[99] Lee W., Lee V., Polio S., Keegan P., Lee J.-H., Fischer K., Park J.-K., Yoo S.-S. (2010). On-demand three-dimensional freeform fabrication of multi-layered hydrogel scaffold with fluidic channels. Biotechnol. Bioeng..

[100] Noor N., Shapira A., Edri R., Gal I., Wertheim L., Dvir T. (2019). 3D printing of personalized thick and perfusable cardiac patches and hearts. Adv. Sci..

[101] Stillitano F., Turnbull I.C., Karakikes I., Nonnenmacher M., Backeris P., Hulot J.-S., Kranias E.G., Hajjar R.J., Costa K.D. (2016). Genomic correction of familial cardiomyopathy in human engineered cardiac tissues. Eur. Heart J..

[102] Ma Z., Huebsch N., Koo S., Mandegar M.A., Siemons B., Boggess S., Conklin B.R., Grigoropoulos C.P., Healy K.E. (2018). Contractile deficits in engineered cardiac microtissues as a result of MYBPC3 deficiency and mechanical overload. Nat. Biomed. Eng..

[103] Parrag I.C., Zandstra P.W., Woodhouse K.A. (2012). Fiber alignment and coculture with fibroblasts improves the differentiated phenotype of murine embryonic stem cell-derived cardiomyocytes for cardiac tissue engineering. Biotechnol. Bioeng..

[104] Weeke-Klimp A., Bax N.A.M., Winter E.M., Vrolijk J., Plantinga J., Maas S., Brinker M., Mahtab E.A.F., Gittenberger-de Groot A.C., van Luyn M.J.A., Harmsen M.C., Lie-Venema H., Anna Rita Bellu (2010). Epicardium-derived cells enhance proliferation, cellular maturation and alignment of cardiomyocytes. J. Mol. Cell. Cardiol..

[105] Murphy J.F., Mayourian J., Stillitano F., Munawar S., Broughton K.M., Agullo-Pascual E., Sussman M.A., Hajjar R.J., Costa K.D., Turnbull I.C. (2019). Adult human cardiac stem cell supplementation effectively increases contractile function and maturation in human engineered cardiac tissues. Stem Cell Res. Ther..

[106] Kitsara M., Agbulut O., Kontziampasis D., Chen Y., Menasché P. (2017). Fibers for hearts: a critical review on electrospinning for cardiac tissue engineering. Acta Biomater..

[107] Huyer L.D., Montgomery M., Zhao Y., Xiao Y., Conant G., Korolj A., Radisic M. (2016).

[108] Annabi N., Nichol J.W., Zhong X., Ji C., Koshy S., Khademhosseini A., Dehghani F. (2010). Controlling the porosity and microarchitecture of hydrogels for tissue engineering. Tissue Eng. B Rev..

[109] Capulli A.K., MacQueen L.A., Sheehy S.P., Parker K.K. (2016). Fibrous scaffolds for building hearts and heart parts. Adv. Drug Deliv. Rev..

[110] Zimmermann W.-H., Melnychenko I., Wasmeier G., Didié M., Naito H., Nixdorff U., Hess A., Budinsky L., Brune K., Michaelis B., Dhein S., Schwoerer A., Ehmke H., Eschenhagen T. (2006). Engineered heart tissue grafts improve systolic and diastolic function in infarcted rat hearts. Nat. Med..

[111] Chung C., Burdick J.A. (2009). Influence of three-dimensional hyaluronic acid microenvironments on mesenchymal stem cell chondrogenesis. Tissue Eng..

[112] Entcheva E., Bien H., Yin L., Chung C.-Y., Farrell M., Kostov Y. (2004). Functional cardiac cell constructs on cellulose-based scaffolding. Biomaterials.

[113] Black L.D., Meyers J.D., Weinbaum J.S., Shvelidze Y.A., Tranquillo R.T. (2009). Cell-induced alignment augments twitch force in fibrin gel–based engineered myocardium via gap junction modification. Tissue Eng..

[114] Duan Y., Liu Z., O'Neill J., Wan L.Q., Freytes D.O., Vunjak-Novakovic G. (2011). Hybrid gel composed of native heart matrix and collagen induces cardiac differentiation of human embryonic stem cells without supplemental growth factors. J. Cardiovasc. Transl. Res..

[115] Kang P.-L., Chen C.-H., Chen S.Y., Wu Y.-J., Lin C.Y., Lin F.-H., Kuo S.M. (2013). Nano-sized collagen I molecules enhanced the differentiation of rat mesenchymal stem cells into cardiomyocytes: enhancement of nano-sized collagen I molecules. J. Biomed. Mater. Res..

[116] Helary C., Abed A., Mosser G., Louedec L., Meddahi-Pellé A., Giraud-Guille M.M. (2011). Synthesis and in vivo integration of improved concentrated collagen hydrogels. J. Tissue Eng. Regen. Med..

[117] Mosser G., Anglo A., Helary C., Bouligand Y., Giraud-Guille M.-M. (2006). Dense tissue-like collagen matrices formed in cell-free conditions. Matrix Biol..

[118] Lv J., Liu W., Shi G., Zhu F., He X., Zhu Z., Chen H. (2019). Human cardiac extracellular matrix-chitosan-gelatin composite scaffold and its endothelialization. Exp. Ther. Med..

[119] Bejleri D., Streeter B.W., Nachlas A.L.Y., Brown M.E., Gaetani R., Christman K.L., Davis M.E. (2018). A bioprinted cardiac patch composed of cardiac‐specific extracellular matrix and progenitor cells for heart repair. Adv. Healthc. Mater..

[120] Umashankar P.R., Sabareeswaran A., Shenoy S.J. (2017). Long-term healing of mildly cross-linked decellularized bovine pericardial aortic patch: long-term healing OF mildly cross-linked bovine pericardium. J. Biomed. Mater. Res. B Appl. Biomater..

[121] Serna J., Florez S., Talero V., Briceño J., Muñoz-Camargo C., Cruz J. (2019). Formulation and characterization of a SIS-based photocrosslinkable bioink. Polymers.

[122] Elder S., Pinheiro A., Young C., Smith P., Wright E. (2017). Evaluation of genipin for stabilization of decellularized porcine cartilage: genipin stabilization OF acellular cartilage. J. Orthop. Res..

[123] Xu Y., Hu Y., Liu C., Yao H., Liu B., Mi S. (2018). A novel strategy for creating tissue-engineered biomimetic blood vessels using 3D bioprinting technology. Materials.

[124] Kolesky D.B., Truby R.L., Gladman A.S., Busbee T.A., Homan K.A., Lewis J.A. (2014). 3D bioprinting of vascularized, heterogeneous cell-laden tissue constructs. Adv. Mater..

[125] Chan B.P., So K.-F. (2005). Photochemical crosslinking improves the physicochemical properties of collagen scaffolds. J. Biomed. Mater. Res..

[126] Kai D., Prabhakaran M.P., Jin G., Ramakrishna S. (2011). Guided orientation of cardiomyocytes on electrospun aligned nanofibers for cardiac tissue engineering. J. Biomed. Mater. Res. B Appl. Biomater..

[127] Kang H.-W., Lee S.J., Ko I.K., Kengla C., Yoo J.J., Atala A. (2016). A 3D bioprinting system to produce human-scale tissue constructs with structural integrity. Nat. Biotechnol..

[128] Guarnieri D., De Capua A., Ventre M., Borzacchiello A., Pedone C., Marasco D., Ruvo M., Netti P.A. (2010). Covalently immobilized RGD gradient on PEG hydrogel scaffold influences cell migration parameters. Acta Biomater..

[129] Hitscherich P., Wu S., Gordan R., Xie L.-H., Arinzeh T., Lee E.J. (2016). The effect of PVDF-TrFE scaffolds on stem cell derived cardiovascular cells: cardiovascular cells on PVDF-TrFE scaffolds. Biotechnol. Bioeng..

[130] Hosseinkhani H., Hosseinkhani M., Hattori S., Matsuoka R., Kawaguchi N. (2010). Micro and nano-scale in vitro 3D culture system for cardiac stem cells. J. Biomed. Mater. Res..

[131] Li J., Minami I., Shiozaki M., Yu L., Yajima S., Miyagawa S., Shiba Y., Morone N., Fukushima S., Yoshioka M., Li S., Qiao J., Li X., Wang L., Kotera H., Nakatsuji N., Sawa Y., Chen Y., Liu L. (2017). Human pluripotent stem cell-derived cardiac tissue-like constructs for repairing the infarcted myocardium. Stem Cell Rep..

[132] Schwan J., Kwaczala A.T., Ryan T.J., Bartulos O., Ren Y., Sewanan L.R., Morris A.H., Jacoby D.L., Qyang Y., Campbell S.G. (2016). Anisotropic engineered heart tissue made from laser-cut decellularized myocardium. Sci. Rep..

[133] Singelyn J.M., Sundaramurthy P., Johnson T.D., Schup-Magoffin P.J., Hu D.P., Faulk D.M., Wang J., Mayle K.M., Bartels K., Salvatore M., Kinsey A.M., DeMaria A.N., Dib N., Christman K.L. (2012). Catheter-deliverable hydrogel derived from decellularized ventricular extracellular matrix increases endogenous cardiomyocytes and preserves cardiac function post-myocardial infarction. J. Am. Coll. Cardiol..

[134] Rajabi-Zeleti S., Jalili-Firoozinezhad S., Azarnia M., Khayyatan F., Vahdat S., Nikeghbalian S., Khademhosseini A., Baharvand H., Aghdami N. (2014). The behavior of cardiac progenitor cells on macroporous pericardium-derived scaffolds. Biomaterials.

[135] Lu T.-Y., Lin B., Kim J., Sullivan M., Tobita K., Salama G., Yang L. (2013). Repopulation of decellularized mouse heart with human induced pluripotent stem cell-derived cardiovascular progenitor cells. Nat. Commun..

[136] Godier-Furnemont A.F.G., Martens T.P., Koeckert M.S., Wan L., Parks J., Arai K., Zhang G., Hudson B., Homma S., Vunjak-Novakovic G. (2011). Composite scaffold provides a cell delivery platform for cardiovascular repair. Proc. Natl. Acad. Sci. Unit. States Am..

[137] Johnson T.D., Hill R.C., Dzieciatkowska M., Nigam V., Behfar A., Christman K.L., Hansen K.C. (2016). Quantification of decellularized human myocardial matrix: a comparison of six patients. PROTEOMICS - Clin. Appl..

[138] Grover G.N., Rao N., Christman K.L. (2014). Myocardial matrix–polyethylene glycol hybrid hydrogels for tissue engineering. Nanotechnology.

[139] Tulloch N.L., Muskheli V., Razumova M.V., Korte F.S., Regnier M., Hauch K.D., Pabon L., Reinecke H., Murry C.E. (2011). Growth of engineered human myocardium with mechanical loading and vascular coculture. Circ. Res..

[140] Engler A.J., Carag-Krieger C., Johnson C.P., Raab M., Tang H.-Y., Speicher D.W., Sanger J.W., Sanger J.M., Discher D.E. (2008). Embryonic cardiomyocytes beat best on a matrix with heart-like elasticity: scar-like rigidity inhibits beating. J. Cell Sci..

[141] Heras-Bautista C.O., Mikhael N., Lam J., Shinde V., Katsen-Globa A., Dieluweit S., Molcanyi M., Uvarov V., Jütten P., Sahito R.G.A., Mederos-Henry F., Piechot A., Brockmeier K., Hescheler J., Sachinidis A., Pfannkuche K. (2019). Cardiomyocytes facing fibrotic conditions Re-express extracellular matrix transcripts. Acta Biomater..

[142] Klotz B.J., Lim K.S., Chang Y.X., Soliman B.G., Pennings I., Melchels F.P.W., Woodfield T.B.F., Rosenberg A.J.W.P., Malda J., Gawlitta D. (2018). Engineering of a complex bone tissue model with endothelialised channels and capillary-like networks. Eur. Cell. Mater..

[143] Lu, J. X.; Flautre, B.; Anselme, K.; Hardouin, P.; Gallur, A.; Descamps, M.; Thierry, B. Role of Interconnections in Porous Bioceramics on Bone Recolonization in Vitro and in Vivo. vol. 10. 10.1023/a:1008973120918. 15347932

[144] Chan B.P., Leong K.W. (2008). Scaffolding in tissue engineering: general approaches and tissue-specific considerations. Eur. Spine J..

[145] Cashman T.J., Josowitz R., Johnson B.V., Gelb B.D., Costa K.D. (2016). Human engineered cardiac tissues created using induced pluripotent stem cells reveal functional characteristics of BRAF-mediated hypertrophic cardiomyopathy. PloS One.

[146] Masumoto H., Nakane T., Tinney J.P., Yuan F., Ye F., Kowalski W.J., Minakata K., Sakata R., Yamashita J.K., Keller B.B. (2016). The myocardial regenerative potential of three-dimensional engineered cardiac tissues composed of multiple human IPS cell-derived cardiovascular cell lineages. Sci. Rep..

[147] Sarig-Nadir O., Livnat N., Zajdman R., Shoham S., Seliktar D. (2009). Laser photoablation of guidance microchannels into hydrogels directs cell growth in three dimensions. Biophys. J..

[148] Zhang S. (2003). Fabrication of novel biomaterials through molecular self-assembly. Nat. Biotechnol..

[149] Schneider A., Garlick J.A., Egles C. (2008). Self-assembling peptide nanofiber scaffolds accelerate wound healing. PloS One.

[150] Rexeisen E.L., Fan W., Pangburn T.O., Taribagil R.R., Bates F.S., Lodge T.P., Tsapatsis M., Kokkoli E. (2010). Self-assembly of fibronectin mimetic peptide-amphiphile nanofibers. Langmuir.

[151] Ban K., Park H.-J., Kim S., Andukuri A., Cho K.-W., Hwang J.W., Cha H.J., Kim S.Y., Kim W.-S., Jun H.-W., Yoon Y.-S. (2014). Cell therapy with embryonic stem cell-derived cardiomyocytes encapsulated in injectable nanomatrix gel enhances cell engraftment and promotes cardiac repair. ACS Nano.

[152] Christoph S., Kwiatoszynski J., Coradin T., Fernandes F.M. (2016). Cellularized cellular solids via freeze-casting: cellularized cellular solids via freeze-casting. Macromol. Biosci..

[153] Rieu C., Parisi C., Mosser G., Haye B., Coradin T., Fernandes F.M., Trichet L. (2019). Topotactic fibrillogenesis of freeze-cast microridged collagen scaffolds for 3D cell culture. ACS Appl. Mater. Interfaces.

[154] Vepari C., Kaplan D.L. (2007). Silk as a biomaterial. Prog. Polym. Sci..

[155] Subia B., Kundu J., S C., Eberli D. (2010). Biomaterial scaffold fabrication techniques for potential tissue engineering applications. Tissue Engineering.

[156] Tadic D., Beckmann F., Schwarz K., Epple M. (2004). A novel method to produce hydroxyapatite objects with interconnecting porosity that avoids sintering. Biomaterials.

[157] Moore M.J., Jabbari E., Ritman E.L., Lu L., Currier B.L., Windebank A.J., Yaszemski M.J. (2004). Quantitative analysis of interconnectivity of porous biodegradable scaffolds with micro-computed tomography. J. Biomed. Mater. Res..

[158] Takagi, S.; Chow, L. C. Formation of Macropores in Calcium Phosphate Cement Implants. vol. 5. 10.1023/a:1008917910468 15348319

[159] Xu H.H.K., Quinn J.B. (2002). Calcium phosphate cement containing resorbable fibers for short-term reinforcement and macroporosity. Biomaterials.

[160] Ganji Y., Li Q., Quabius E.S., Böttner M., Selhuber-Unkel C., Kasra M. (2016). Cardiomyocyte behavior on biodegradable polyurethane/gold nanocomposite scaffolds under electrical stimulation. Mater. Sci. Eng. C.

[161] Chrobak K.M., Potter D.R., Tien J. (2006). formation of perfused, functional microvascular tubes in vitro. Microvasc. Res..

[162] Mori N., Morimoto Y., Takeuchi S. (2017). Skin integrated with perfusable vascular channels on a chip. Biomaterials.

[163] Sargeant T.D., Guler M.O., Oppenheimer S.M., Mata A., Satcher R.L., Dunand D.C., Stupp S.I. (2008). Hybrid bone implants: self-assembly of peptide amphiphile nanofibers within porous titanium. Biomaterials.

[164] Karageorgiou V., Kaplan D. (2005). Porosity of 3D biomaterial scaffolds and osteogenesis. Biomaterials.

[165] Murphy S.V., Atala A. (2014). 3D bioprinting of tissues and organs. Nat. Biotechnol..

[166] Zhang Y.S., Davoudi F., Walch P., Manbachi A., Luo X., Dell'Erba V., Miri A.K., Albadawi H., Arneri A., Li X., Wang X., Dokmeci M.R., Khademhosseini A., Oklu R. (2016). Bioprinted thrombosis-on-a-chip. Lab Chip.

[167] Lee A., Hudson A.R., Shiwarski D.J., Tashman J.W., Hinton T.J., Yerneni S., Bliley J.M., Campbell P.G., Feinberg A.W. (2019). 3D bioprinting of collagen to rebuild components of the human heart. Science.

[168] Datta P., Ayan B., Ozbolat I.T. (2017). Bioprinting for vascular and vascularized tissue biofabrication. Acta Biomater..

[169] McCormack A., Highley C.B., Leslie N.R., Melchels F.P.W. (2020). 3D printing in suspension baths: keeping the promises of bioprinting afloat. Trends Biotechnol..

[170] Do A.-V., Khorsand B., Geary S.M., Salem A.K. (2015). 3D printing of scaffolds for tissue regeneration applications. Adv. Healthc. Mater..

[171] Gaetani R., Doevendans P.A., Metz C.H.G., Alblas J., Messina E., Giacomello A., Sluijter J.P.G. (2012). Cardiac tissue engineering using tissue printing technology and human cardiac progenitor cells. Biomaterials.

[172] Pati F., Jang J., Ha D.-H., Won Kim S., Rhie J.-W., Shim J.-H., Kim D.-H., Cho D.-W. (2014). Printing three-dimensional tissue analogues with decellularized extracellular matrix bioink. Nat. Commun..

[173] Tsukamoto Y., Akagi T., Akashi M. (2020). Vascularized cardiac tissue construction with orientation by layer-by-layer method and 3D printer. Sci. Rep..

[174] Yang X., Pabon L., Murry C.E. (2014). Engineering adolescence: maturation of human pluripotent stem cell–derived cardiomyocytes. Circ. Res..

[175] Han J., Wu Q., Xia Y., Wagner M.B., Xu C. (2016). Cell alignment induced by anisotropic electrospun fibrous scaffolds alone has limited effect on cardiomyocyte maturation. Stem Cell Res..

[176] Zarkoob S., Eby R.K., Reneker D.H., Hudson S.D., Ertley D., Adams W.W. (2004). Structure and morphology of electrospun silk nanofibers. Polymer.

[177] Traversa E., Mecheri B., Mandoli C., Soliman S., Rinaldi A., Licoccia S., Forte G., Pagliari F., Pagliari S., Carotenuto F., Minieri M., Di Nardo P. (2008). Tuning hierarchical architecture of 3D polymeric scaffolds for cardiac tissue engineering. J. Exp. Nanosci..

[178] Rockwood D.N., Akins R.E., Parrag I.C., Woodhouse K.A., Rabolt J.F. (2008). Culture on electrospun Polyurethane scaffolds decreases atrial natriuretic peptide expression by cardiomyocytes in vitro. Biomaterials.

[179] Ohkawa K., Cha D., Kim H., Nishida A., Yamamoto H. (2004). Electrospinn. Chitosan. Macromol. Rapid Commun..

[180] Matthews J.A., Wnek G.E., Simpson D.G., Bowlin G.L. (2002). Electrospinning of collagen nanofibers. Biomacromolecules.

[181] Joanne P., Kitsara M., Boitard S.-E., Naemetalla H., Vanneaux V., Pernot M., Larghero J., Forest P., Chen Y., Menasché P., Agbulut O. (2016). Nanofibrous clinical-grade collagen scaffolds seeded with human cardiomyocytes induces cardiac remodeling in dilated cardiomyopathy. Biomaterials.

[182] Picaut L., Trichet L., Ronsin O., Haye B., Génois I., Baumberger T., Mosser G. (2018). Pure dense collagen threads from extrusion to fibrillogenesis stability. Biomed. Phys. Eng. Express.

[183] Schwab A., Hélary C., Richards R.G., Alini M., Eglin D., D'Este M. (2020). Tissue mimetic hyaluronan bioink containing collagen fibers with controlled orientation modulating cell migration and alignment. Mater. Today Biol..

[184] Kim H., Jang J., Park J., Lee K.-P., Lee S., Lee D.-M., Kim K.H., Kim H.K., Cho D.-W. (2019). Shear-induced alignment of collagen fibrils using 3D cell printing for corneal stroma tissue engineering. Biofabrication.

[185] Guo T., Ringel J.P., Lim C.G., Bracaglia L.G., Noshin M., Baker H.B., Powell D.A., Fisher J.P. (2018). Three dimensional extrusion printing induces polymer molecule alignment and cell organization within engineered cartilage: 3D printing for cellular organization IN engineered cartilage. J. Biomed. Mater. Res..

[186] Yuan Ye K., Sullivan K.E., Black L.D. (2011). Encapsulation of cardiomyocytes in a fibrin hydrogel for cardiac tissue engineering. JoVE.

[187] Worcester D.L. (1978). Structural origins of diamagnetic anisotropy in proteins. Proc. Natl. Acad. Sci. Unit. States Am..

[188] Torbet J., Malbouyres M., Builles N., Justin V., Roulet M., Damour O., Oldberg Å., Ruggiero F., Hulmes D.J.S. (2007). Orthogonal scaffold of magnetically aligned collagen lamellae for corneal stroma reconstruction. Biomaterials.

[189] Guo C., Kaufman L.J. (2007). Flow and magnetic field induced collagen alignment. Biomaterials.

[190] Chen S., Hirota N., Okuda M., Takeguchi M., Kobayashi H., Hanagata N., Ikoma T. (2011). Microstructures and rheological properties of Tilapia fish-scale collagen hydrogels with aligned fibrils fabricated under magnetic fields. Acta Biomater..

[191] Hinson J.T., Chopra A., Lowe A., Sheng C.C., Gupta R.M., Kuppusamy R., O'Sullivan J., Rowe G., Wakimoto H., Gorham J., Burke M.A., Zhang K., Musunuru K., Gerszten R.E., Wu S.M., Chen C.S., Seidman J.G., Seidman C.E. (2016). Integrative analysis of PRKAG2 cardiomyopathy IPS and microtissue models identifies AMPK as a regulator of metabolism, survival, and fibrosis. Cell Rep..

[192] Makarenko I., Opitz C.A., Leake M.C., Neagoe C., Kulke M., Gwathmey J.K., del Monte F., Hajjar R.J., Linke W.A. (2004). Passive stiffness changes caused by upregulation of compliant titin isoforms in human dilated cardiomyopathy hearts. Circ. Res..

[193] Sala L., van Meer B.J., Tertoolen L.G.J., Bakkers J., Bellin M., Davis R.P., Denning C., Dieben M.A.E., Eschenhagen T., Giacomelli E., Grandela C., Hansen A., Holman E.R., Jongbloed M.R.M., Kamel S.M., Koopman C.D., Lachaud Q., Mannhardt I., Mol M.P.H., Mosqueira D., Orlova V.V., Passier R., Ribeiro M.C., Saleem U., Smith G.L., Burton F.L., Mummery C.L. (2018). MUSCLEMOTION: a versatile open software tool to quantify cardiomyocyte and cardiac muscle contraction in vitro and in vivo. Circ. Res..

[194] Gepstein L., Ding C., Rehemedula D., Wilson E.E., Yankelson L., Caspi O., Gepstein A., Huber I., Olgin J.E. (2010). In Vivo assessment of the electrophysiological integration and arrhythmogenic risk of myocardial cell transplantation strategies. Stem Cell..

[195] Itzhaki I., Maizels L., Huber I., Zwi-Dantsis L., Caspi O., Winterstern A., Feldman O., Gepstein A., Arbel G., Hammerman H., Boulos M., Gepstein L. (2011). Modelling the long QT syndrome with induced pluripotent stem cells. Nature.

[196] Tian B., Liu J., Dvir T., Jin L., Tsui J.H., Qing Q., Suo Z., Langer R., Kohane D.S., Lieber C.M. (2012). Macroporous nanowire nanoelectronic scaffolds for synthetic tissues. Nat. Mater..

[197] Feiner R., Engel L., Fleischer S., Malki M., Gal I., Shapira A., Shacham-Diamand Y., Dvir T. (2016). Engineered hybrid cardiac patches with multifunctional electronics for online monitoring and regulation of tissue function. Nat. Mater..

[198] Joshi J., Rubart M., Zhu W. (2020). Optogenetics: background, methodological advances and potential applications for cardiovascular research and medicine. Front. Bioeng. Biotechnol..

[199] Yan Zhuge, Patlolla B., Ramakrishnan C., Beygui R.E., Zarins C.K., Deisseroth K., Kuhl E., Abilez O.J. (2014). Human pluripotent stem cell tools for cardiac optogenetics. 2014 36th Annual International Conference of the IEEE Engineering in Medicine and Biology Society.

[200] Park S.A., Lee S.-R., Tung L., Yue D.T. (2015). Optical mapping of optogenetically shaped cardiac action potentials. Sci. Rep..

[201] Breckwoldt K., Letuffe-Brenière D., Mannhardt I., Schulze T., Ulmer B., Werner T., Benzin A., Klampe B., Reinsch M.C., Laufer S., Shibamiya A., Prondzynski M., Mearini G., Schade D., Fuchs S., Neuber C., Krämer E., Saleem U., Schulze M.L., Rodriguez M.L., Eschenhagen T., Hansen A. (2017). Differentiation of cardiomyocytes and generation of human engineered heart tissue. Nat. Protoc..

[202] Lin Gisela, Palmer R.E., Pister K.S.J., Roos K.P. (2001). Miniature heart cell force transducer system implemented in MEMS technology. IEEE Trans. Biomed. Eng..

[203] Gaitas A., Malhotra R., Li T., Herron T., Jalife J. (2015). A device for rapid and quantitative measurement of cardiac myocyte contractility. Rev. Sci. Instrum..

[204] Ribeiro M.C., Tertoolen L.G., Guadix J.A., Bellin M., Kosmidis G., D'Aniello C., Monshouwer-Kloots J., Goumans M.-J., Wang Y., Feinberg A.W., Mummery C.L., Passier R. (2015). Functional maturation of human pluripotent stem cell derived cardiomyocytes in vitro – correlation between contraction force and electrophysiology. Biomaterials.

[205] Chang W.-T., Yu D., Lai Y.-C., Lin K.-Y., Liau I. (2013). Characterization of the mechanodynamic response of cardiomyocytes with atomic force microscopy. Anal. Chem..

[206] Wang G., McCain M.L., Yang L., He A., Pasqualini F.S., Agarwal A., Yuan H., Jiang D., Zhang D., Zangi L., Geva J., Roberts A.E., Ma Q., Ding J., Chen J., Wang D.-Z., Li K., Wang J., Wanders R.J.A., Kulik W., Vaz F.M., Laflamme M.A., Murry C.E., Chien K.R., Kelley R.I., Church G.M., Parker K.K., Pu W.T. (2014). Modeling the mitochondrial cardiomyopathy of barth syndrome with induced pluripotent stem cell and heart-on-chip technologies. Nat. Med..

